# Supramolecular Nanopatterns of Molecular Spoked Wheels with Orthogonal Pillars: The Observation of a Fullerene Haze

**DOI:** 10.1002/anie.202111869

**Published:** 2021-11-23

**Authors:** Georgiy Poluektov, Tristan J. Keller, Anna Jochemich, Anna Krönert, Ute Müller, Sebastian Spicher, Stefan Grimme, Stefan‐S. Jester, Sigurd Höger

**Affiliations:** ^1^ Kekulé-Institut für Organische Chemie und Biochemie Rheinische Friedrich-Wilhelms-Universität Bonn Gerhard-Domagk-Str. 1 53121 Bonn Germany; ^2^ Mulliken Center for Theoretical Chemistry Rheinische Friedrich-Wilhelms-Universität Bonn Beringstr. 4 53115 Bonn Germany

**Keywords:** molecular modeling, molecular pillars, molecular spoked wheels, nanostructures, scanning probe microscopy

## Abstract

Molecular spoked wheels with intraannular functionalizable pillars are synthesized in a modular approach. The functionalities at their ends are variable, and a propargyl alcohol, a [6,6]‐phenyl‐C61‐butyrate, and a perylene monoimide are investigated. All compounds form two‐dimensional crystals on highly oriented pyrolytic graphite at the solid–liquid interface. As determined by submolecularly resolved scanning tunneling microscopy, the pillars adopt equilibrium distances of 6.0 nm. The fullerene has a residual mobility, limited by the length of the flexible connector unit. The experimental results are supported and rationalized by molecular dynamics simulations. These also show that, in contrast, the more rigidly attached perylene monoimide units remain oriented along the surface normal and maintain a smallest distance of 2 nm above the graphite substrate. The robust packing concept also holds for cocrystals with molecular hexagons that expand the pillar–pillar distances by 15 % and block unspecific intercalation.

## Graphical abstract

A modular synthetic approach paves the way to nanoscale platform molecules for supramolecular graphite surface patterns at the solid–liquid interface. While a perylene dye bound at the end of the pillar remains fixed above the molecule center and points into solution, a fullerene ester bound at the same position performs a tumbling motion that translates as a “haze” in the otherwise submolecularly resolved images.

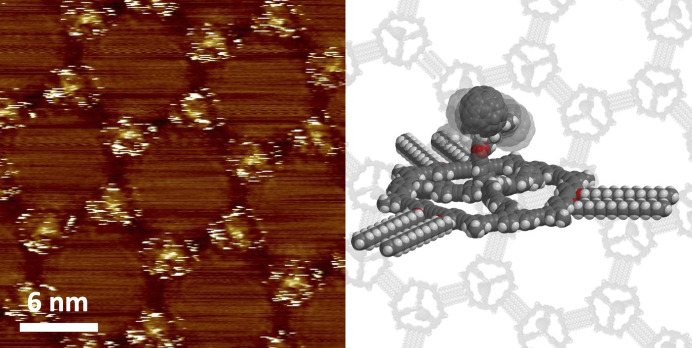

The surface of each solid, liquid, or soft object is the region where it interacts with its environment. A surface can be characterized by its structure,^[1]^ which can be smooth with a high reflectivity (e.g. for metals) or corrugated with reduced friction (“shark skin effect”).^[2]^ Although connected with a high visibility, the specific chemical nature of the surface is of minor importance as long as it is stable in the given environment, meaning, in principle the surface material is exchangeable. On the other hand, a surface can be characterized by its function, that is, its ability to fulfill a specific task. Here, the visibility may be limited, but the specific chemical composition is of utmost importance, and therefore materials are generally not exchangeable. Such properties can be achieved with the topmost layer of the volume phase or with a specific adlayer. Examples include heterogeneous olefin polymerization catalysts on a solid support, or the spike protein on a virus surface.^[3]^ Such functionalities must sometimes be spatially and laterally decoupled from the surface and each other, respectively, to fulfill their task.[^4^, ^5^]

Of particular interest in this context are artificial platform structures carrying functionalizable pillars pointing out of the molecule plane, accessible in a modular synthesis. After adsorption of the molecules from solution or gas phase to a solid surface, these pillar units are directed along the surface normal, point towards the supernatant solution phase, gas phase, or vacuum, and therefore allow for electronic or mechanical decoupling of adjunct units from the direct contact with the surface. Literature examples include triazatriangulenium (TATA) based platforms with various horizontal and vertical spacers^[6a]^ or tripods with pyrene or thiol adhesives.[^6b^, ^6c^, ^6d^] However, the number of examples that follow a modular synthetic design concept and give predictable two‐dimensional (2D) patterns, which are not influenced by the specific unit that points into the volume phase, is yet limited. Moreover, both synthetic effort and limited persistence length make it even more difficult to address specific periodically arranged positions with lateral spacing in the range of several nanometers.

Adjustable packings between rigid oblate molecules in 2D can be achieved by the attachment of alkyl/alkoxy chains that decorate the surface of highly oriented pyrolytic graphite (HOPG) in a reliable way: They orient along its main axes, pack densely, and thereby drive the adsorption of the molecular species and thus the formation of 2D crystals.^[7]^ Sophisticated approaches like shape‐persistency and shape‐complementarity even allow the co‐adsorption of two or more components,^[8]^ leading to highly complex 2D nanopatterns.^[9]^ Such structures can be investigated by scanning tunneling microscopy (STM), providing submolecular (and often atomic) resolution. STM is used not only to investigate planar molecules, but also bilayers^[10]^ or molecules with a distinct expansion into the third dimension (3D).[^6a^, ^6c^, ^6d^, ^11^]

In our previous work, we focused mainly on the structuring of the graphite surface, including homo‐ as well as coadsorptions of macrocycles,^[8b]^ molecular spoked wheels (MSWs),[^10b^, ^12^] and also highly strained bicyclophanes with vertical extensions along the surface normal.^[11]^ The sizes of the latter are adjustable, and the pillars at the intraannular bridges are separated up to 2 nm, although the other parts of the molecules are densely packed.

Here, we describe arylene‐alkynylenes **1**–**3** that combine the predictable and reliable pattern formation of alkyl substituted MSWs with the molecular platform approach. Specifically, our structures are based on an MSW with a central orthogonally arranged rigid pillar, and are accessible by a modular synthetic approach. These molecules allow for adjustable pillar–pillar distances, heights, and variable functionalization, and a propargyl alcohol (**1**), a fullerene derivative (**2**), as well as a perylene monoimide (**3**) are investigated (Figure 1).

**Figure 1:**
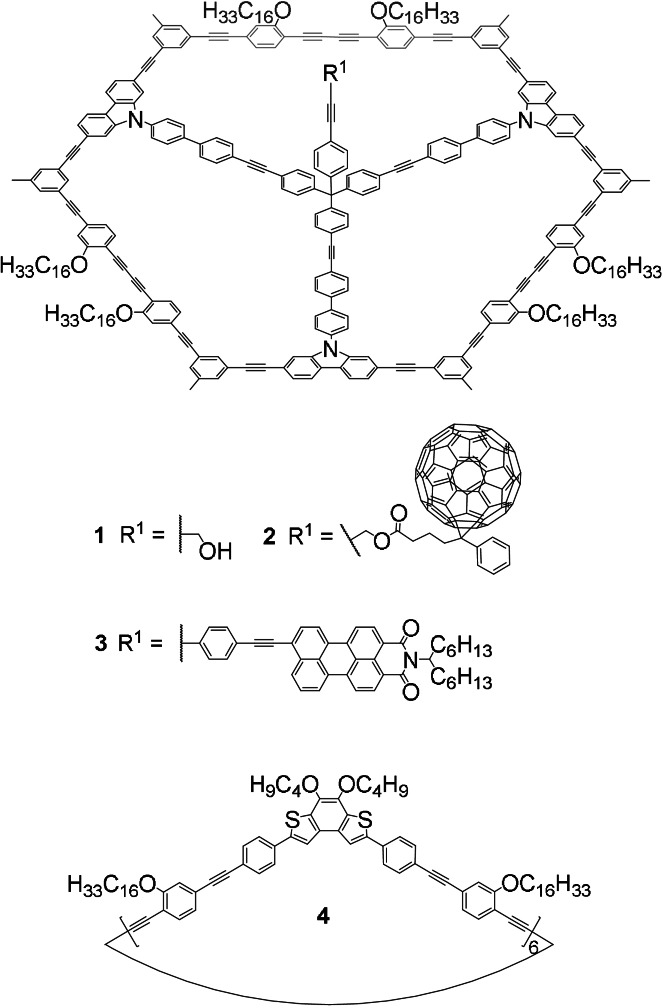
Chemical structures of molecular spoked wheels **1**–**3** with pillars carrying a propargyl alcohol (**1**), a [6,6]‐phenyl‐C61‐butyrate (**2**), and a perylene monoimide unit (**3**), as well as arylene‐alkynylene hexagon (**4**).

For the syntheses of the target structures (Scheme 1), the spoke/rim segments **5** were coupled to the hub molecules **6 a** and **6 b** via Sonogashira reaction and gave **7 a** and **7 b** in yields of 78 % and 73 %, respectively. While the hub modules contain a hydroxy or a (protected) acetylene group for further functionalization, the spoke/rim modules contain OC_16_H_33_ side chains that ensure the solubility of the compounds and additionally promote the periodic adsorption of the target structures onto graphite by intermolecular interdigitation. As such, they determine the intermolecular distances between the functionalities of the molecules in the 2D array. After deprotection of the edge acetylenes of **7 a**/**b**, the intramolecular wheel formation of **8 a**/**b** proceeded well to yield 64 % of **1** and 32 % of **9**. As examples of the functionalization at the pillars of the molecules, the esterification of **1** with [6,6]‐phenyl‐C61‐butyric acid (PCBA) to **2** as well as the deprotection of **9** and coupling with an iodoperylene monoimide to **3** were chosen. The synthesis of **4** has been described previously.^[8b]^

**Scheme 1:**
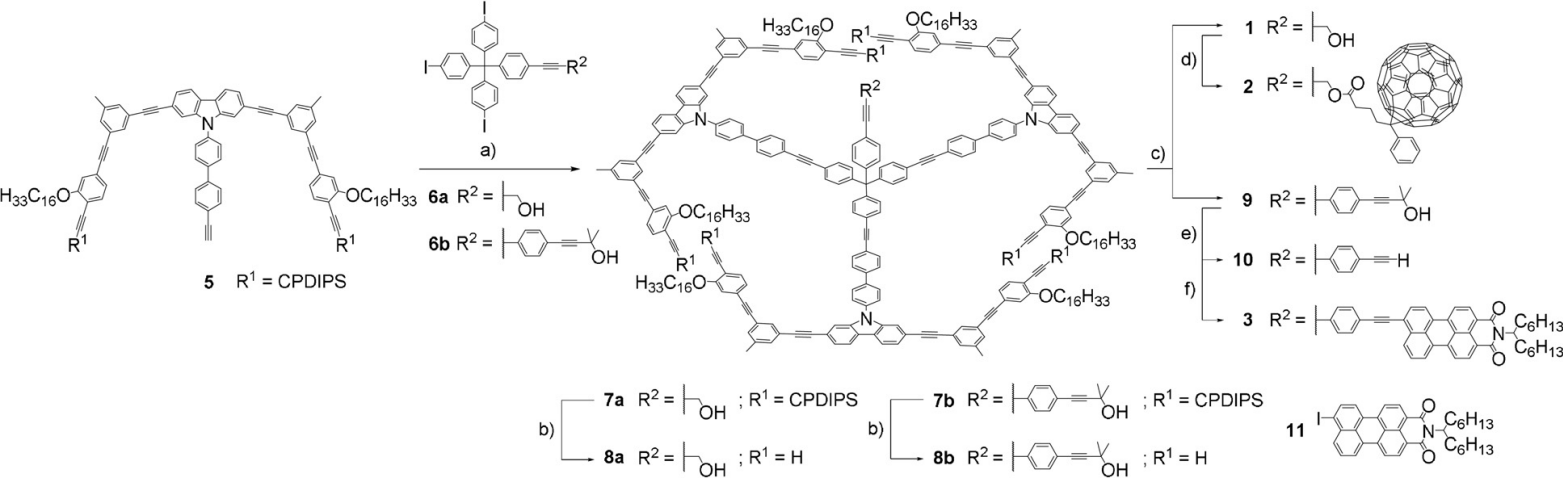
Synthesis of **1**–**3**. a) Pd(PPh_3_)_4_, CuI, THF, piperidine, 50 °C; **7 a**: 20 h, 78 %; **7 b**: 18 h, 73 %; b) 1 m ^*t*^Bu_4_NF in THF, DCM, r.t.; **8 a**: 1 h, 63 %; **8 b**: 3 h, 57 %; c) PdCl_2_(PPh_3_)_2_, CuI, I_2_, THF, HN^*i*^Pr_2_, 50 °C; **1**: 68 h, 64 %; **9**: 60 h, 32 %; d) DMAP, DCC, DCM, r.t., 17 h, 40 %; e) 1 m ^*n*^Bu_4_NOH in MeOH, toluene, 75 °C, 2 h, 62 %; f) Pd(Ph_3_)_4_, CuI, THF, piperidine, r.t., 18 h, 30 %.

Molecular models of **1**–**4** are shown in Figure 2. **1**–**3** consist of *C*_3_‐symmetric (truncated triangular) MSW platforms carrying three pairs of OC_16_H_33_ side chains with chain‐chain distances *w*=8.5 Å and pair‐pair angles (e.g. *γ*(*d*_1_,*d*_3_)) of 120°, designed for effective and stable interdigitation patterns on graphite.[^7^, ^8b^] Three arms of each tetraphenylmethane unit are connected to three carbazole units, while the fourth phenylene unit carries a propargyl alcohol (**1**), a fullerene derivative (**2**), or a perylene monoimide (**3**) moiety sticking out by 1.3 nm, 2.5 nm, and 2.8 nm above the wheel planes, respectively, thus reducing the overall molecular symmetry to *C*_S_.^[13]^ **4** is a *D*_6*h*_‐symmetric (hexagonal) arylene‐alkynylene macrocycle with six pairs of side chains of the same lengths and spacings, but angles (e.g. *γ*(*d*_1_,*d*_2_)) of 60°, thus we predict its complementarity to the platforms of **1**–**3** in 2D nanopatterns.^[8b]^

**Figure 2:**
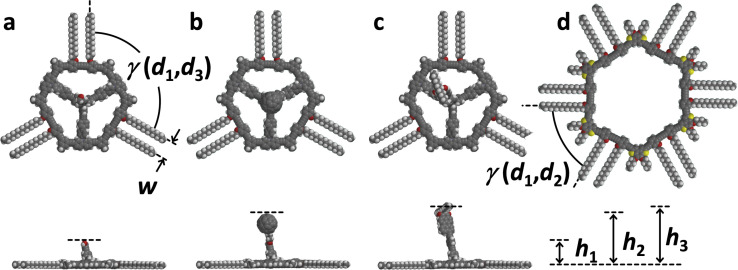
Molecular models of a) **1** (*γ*(*d*_1_,*d*_3_)=120°; *w*=8.5 Å; *h*_**1**_=1.3 nm), b) **2** (*h*_**2**_=2.5 nm), c) **3** (*h*_**3**_=2.8 nm), and d) **4** (*γ*(*d*_1_,*d*_3_)=60°).^[7b]^ Top and side view are given for **1**–**3**.

Self‐assembled monolayers (SAMs) of the pure compounds **1** to **3** and their binary mixtures with **4** at the solid–liquid interface of HOPG and a solution of the respective compound in 1,2,4‐trichlorobenzene (TCB) were investigated by STM. In general, bright and dark image regions are assigned to surface areas of low and high tunneling resistivities, resulting from local densities of state and topographic extensions.^[14]^ At a concentration of *c=*3×10^−7^ m in the supernatant solution phase, **1** forms domains (with sizes of >70^2^ nm^2^, see Supporting Information (SI)) of a chiral honeycomb pattern, to which a unit cell with parameters *a*=*b*=(10.4±0.3) nm and *γ*(*a*,*b*)=(60±2)° containing two molecules with backbone orientations *c*_A_ ∥ *d*_1_ and *c*_B_ with *γ*(*c*_A_,*c*_B_)=60° is indexed (Figure 3 a h^−1^/k/l), and which is oriented relative to the indicated HOPG main axis direction *d*_1_ with *γ*(*a*,*d*_1_)=(25±2)°. Each of the macrocyclic backbones, appearing as bright (nearly circular) truncated triangles, is connected by intermolecularly interdigitating OC_16_H_33_ chains with three adjacent molecules. The latter appear dark and are aligned along the three HOPG main axis directions *d*_1_, *d*_3_, *d*_5_ with orientations of 120° relative to each other. They interdigitate intermolecularly with those of three adjacent molecules, resulting in ABAB side chain interdigitation motifs (cf. Figure 3 k), as it was planned. Within each domain, all innermost side chains relative to the intermolecular nanopores are either oriented in clockwise ((−), Figure 3 k) or counterclockwise (+), fashion.^[15]^ The alkoxy side chain quadruplets and macrocyclic backbone segments surround intermolecular nanopores with a diameter, *D*_1_, of 7.5 nm filled with (microscopically unresolvable) solvent molecules, and some pores (as for example, the one marked by arrow 1 in Figure 3 a) contain weakly visible intercalated molecules of **1** that have a residual mobility due to unspecific interactions with the nanopore walls. The nanopatterns of alkoxy‐substituted MSW scaffolds template periodically assembled propargyl alcohol groups that are clearly visible as bright dots in the centers of each MSW and adopt distances of *a*/√3=(6.0±0.1) nm.^[16a]^ These results stimulated us to investigate **2**, which carries a sterically demanding PCBA ester at its pillar end. After adsorption from a 5×10^−7^ m solution, a chiral honeycomb packing with a domain size of 90^2^ nm^2^ (see SI) is observed, to which a unit cell with parameters *a*=*b*=(10.4±0.3) nm and *γ*(*a*,*b*)=(60±2)° and an additional packing parameter of *γ*(*a*,*d*)=(25±2)°, containing two molecules, is indexed (Figure 3 b/i m^−1^). In other words, **2** and **1** are isomorphous, which reflects the interchangeability of the intraannular units while maintaining the 2D structure, a clear proof of the robustness of the packing concept. In contrast to **1**, however, in the STM image of **2** (at a moderate negative sample bias voltage of *V*_S_=−0.80 V, Figure 3 b) a “haze” of maximally bright scan line segments is superimposed on each backbone position. These features also dominate the image at moderate and small positive sample bias voltages (of +1.10 V and +0.50 V, respectively, cf. Figures 3 c and d).^[17]^ The diameter of the region of their observation of *D*_2_=5 nm appears enormous, considering the relative van der Waals diameter of the fullerene of 1 nm and the MSW backbone of 4 nm. Given the length and rotational degrees of freedom of the butyl ester linker mediated by σ‐bonds, the fullerene is expected to have a tumbling mobility in a 1×10^−23^ L volume in/above the molecular plane. This entails dynamically changing HOMO energy levels of the fullerene, which is therefore visible in a broad bias voltage regime (see Supporting Information). The observation is in contrast to previous STM images of static adlayers in which the fullerene derivatives were adsorbed stationarily^[18]^ or coadsorbed to shape‐persistent macrocycle templates^[19]^ on HOPG. Our results stimulated us to conduct molecular dynamics simulations of **2** adsorbed on graphene cutouts (C_924_H_84_; Figure 4 a‐c) which were performed using a completely automated partially polarizable generic force‐field (GFN‐FF)^[20]^ in a solvent continuum environment with THF as a substitute for TCB (generalized Born surface area (GBSA) solvation model).^[21]^ Starting from the initial geometry (Figure 4 a) of the fullerene oriented along the surface normal, the fullerene‐surface distance collapses and after only 50 ps reaches a final state in which the fullerene occupies one of the three intraannular pores, which serves as a binding pocket (Figure 4 c, see also video file in the Supporting Information). Thus, the interaction between the fullerene and the continuum solvent under quasi‐equilibrium conditions seems to be weaker than with the pocket. Note that an explicit treatment of the solvent might be required in much longer simulations for more decisive conclusions, which is currently not feasible. Moreover, the adsorbed state of the molecule mobility is perturbed by the STM tip, causing random dynamics, akin to the hopping of C_60_ between adjacent lattice sites of trimesic acid template SAMs on HOPG.^[22]^ However, we show with our approach that it is possible to bind a molecule part onto the HOPG surface and still allow a predetermined radius of motion, and to observe such structures with the STM.

**Figure 3:**
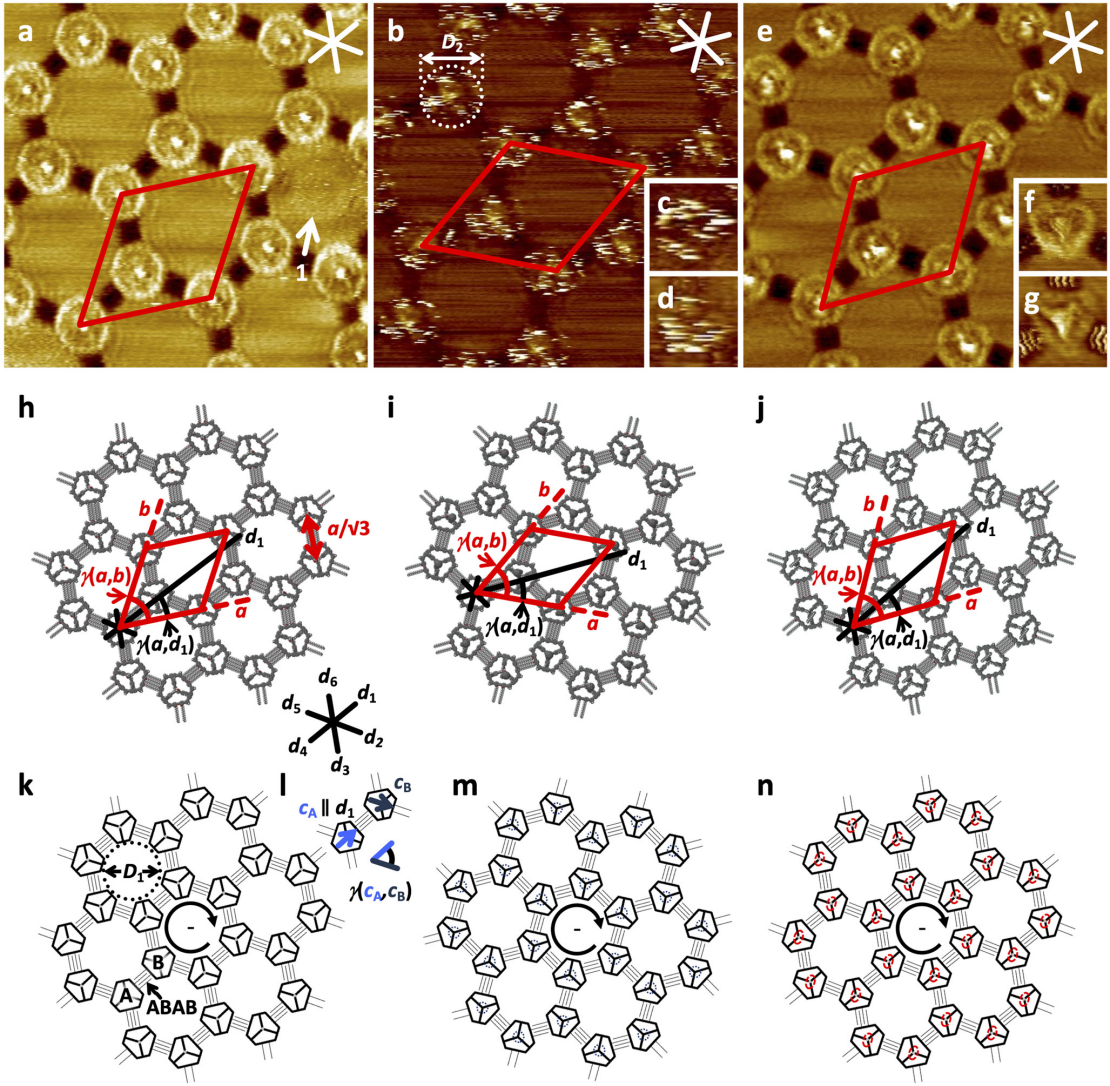
STM images, (supra‐) molecular, and schematic models of self‐assembled monolayers of **1**–**3** at the solid–liquid interface of the respective compound in TCB and HOPG. All samples were thermally annealed for 20 s at 80 °C prior to imaging. a), h), k), l) **1** (30×30 nm^2^, *V*_S_=−0.70 V, *I*_t_=23 pA, *c=*3×10^−7^ m; unit cell: *a*=*b*=(10.4±0.3) nm, *γ*(*a*,*b*)=(60±2)°, additional packing parameters: *γ*(*a*,*d*_1_)=(25±2)°, *a*/√*3*=(6.0±0.1) nm, pore diameter: *D*_1_=(7.5±0.2) nm, ABAB indicates the side chain interdigitation scheme for molecules A and B, that adopt backbone orientations *c*_A_ (defined as bisector) with *c*_A_ ∥ *d*_1_ and neighboring *c*_B_ with *γ*(*c*_A_,*c*_B_)=60°); b)–d), i), m) **2** (b: 30×30 nm^2^, *V*_S_=−0.80 V, *I*_t_=30 pA, *c=*5×10^−7^ m; c: 7.5×7.5 nm^2^ (internal scanner calibration), *V*_S_=+1.10 V, *I*_t_=29 pA, *c=*1×10^−7^ m, d: 7.5×7.5 nm^2^ (internal scanner calibration), *V*_S_=+0.50 V, *I*_t_=9 pA, *c=*1×10^−7^ m; unit cell: *a*=*b*=(10.4±0.3) nm, *γ*(*a*,*b*)=(60±2)°, additional packing parameters: *γ*(*a*,*d*_1_)=(25±2)°); e)–g), j), n) **3** (e: 30×30 nm^2^, *V*_S_=−0.61 V, *I*_t_=21 pA, *c=*1×10^−5^ m; f: 7.5×7.5 nm^2^ (internal scanner calibration), *V*_S_=−0.50 V, *I*_t_=35 pA, *c=*5×10^−7^ m, g: 7.5×7.5 nm^2^ (internal scanner calibration), *V*_S_=+0.50 V, *I*_t_=35 pA, *c=*5×10^−7^ m; unit cell: *a*=*b*=(10.4±0.3) nm, *γ*(*a*,*b*)=(60±2)°, additional packing parameters: *γ*(*a*,*d*_1_)=(25±2)°). Circular arrows in k), m), n) indicate clockwise (−) orientation of the OC_16_H_33_ side chains that border the intermolecular nanopores.^[15]^ All STM images: The red and white (black) lines indicate the unit cells (*a*, *b*) and HOPG main axis directions (*d_n_*), respectively.

**Figure 4:**
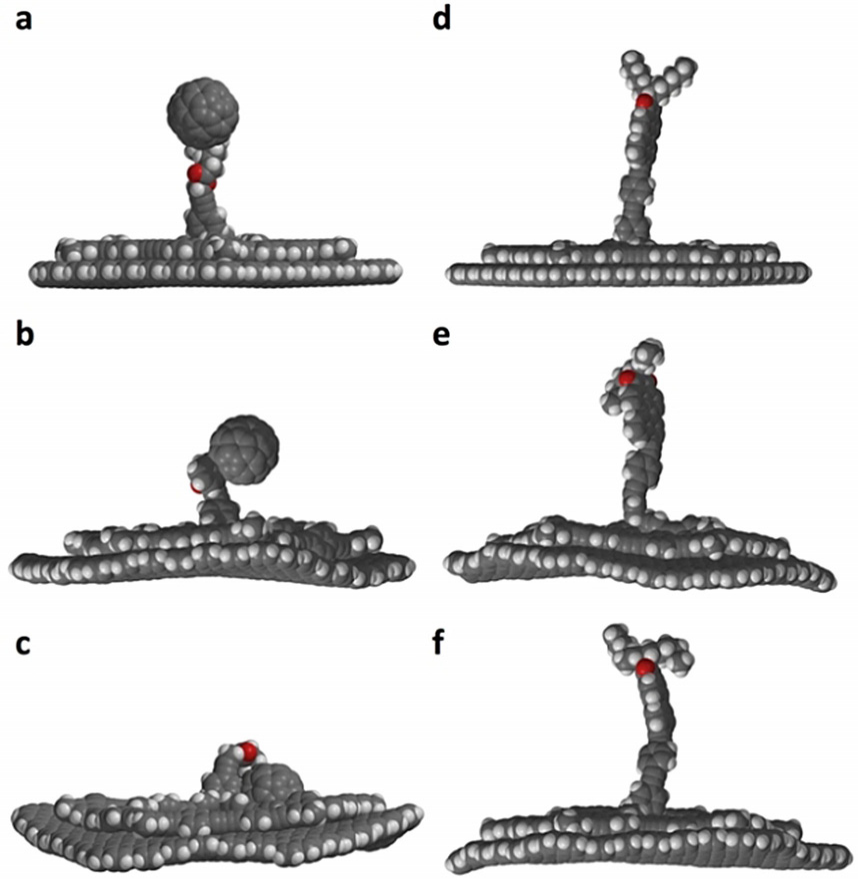
a)–f) Two sequences of still images of molecular dynamics simulations of a)–c) **2** (a: 0 ps, b: 40 ps, c: 1 ns) and d)–f) **3** (d: 0 ps, e: 40 ps, f: 1 ns) on the graphene cutout C_924_H_84_ in a GBSA solvent continuum, obtained by GFN‐FF^[19]^ in THF at 298 K. For video files and additional description see Supporting Information.

Permanent mechanical decoupling of a substituent should be achieved when the functionality is anchored to the pillar while avoiding flexible units. An analogous GFN‐FF/GBSA(THF) MD simulation of **3**, a dialkylated perylene monoimide connected to the MSW platform via a phenylene‐ethynylene linker, on graphenoid C_924_H_84_ (Figure 4 d–f) shows that, in contrast to the fullerene compound, during the entire simulation period (of 1 ns, see video file in the Supporting Information) the linker does not deform to an extent that the perylene unit adsorbs to the HOPG substrate or the macrocycle rim.

However, it was unclear a priori to what extent the different affinities between the planar perylene unit to HOPG and the spherical fullerene unit to HOPG alter the adsorption characteristics of the MSW or even lead to a breakdown of the packing concept. After adsorption of **3** from solution with *c=*1×10^−5^ m (Figure 3 e–g/j/n; to 5×10^−7^ m, see Supporting Information), a chiral honeycomb packing unaltered to those of **1** and **2** with domain sizes of >70^2^ nm^2^ (see SI) is observed, to which (again) a unit cell of *a*=*b*=(10.4±0.3) nm and *γ*(*a*,*b*)=(60±2)° and additional packing parameters of *γ*(*a*,*d*_1_)=(25±2)° are indexed. The macrocyclic backbone segments appear with fainter image contrast at *V*_S_=−0.61 V (Figure 3 e) compared to those of **1** (Figure 3 a), while the central hubs, corresponding to the extended phenylene‐ethynylene pillars appear brighter and still punctate, however with a larger diameter. Our observation of all image parts can be explained by two scenarios: (1) the presence of one HOMO for all aromatic molecule parts, therefore resonant electron tunneling from the substrate through **3** to the tip, or, (2) electronically decoupled MSW backbone and perylene substituted pillar, therefore tunneling from the substrate to separated HOMOs of MSW and pillar to the tip, which requires that both HOMOs are located in the conduction region. Contrary, at small negative (−0.50 V, Figure 3 f) and positive (+ 0.50 V, Figure 3 g) substrate bias voltages, the HOMOs of either MSW backbone or perylene monoimide/pillar are energetically selectively located in the conduction region, respectively, therefore dominate the image contrast to a certain extent (see also Supporting Information). Nevertheless, the high‐resolution STM images of **3** are remarkable: First, the wheel rim (at moderate and higher negative sample bias voltages) is clearly visible despite the central column sticking out by 2.8 nm (i.e. 0.7 × MSW diameter, as determined from the molecular model in Figure 2 c)^[16b]^ from the wheel plane, and second, the 2D packing motif of the wheel is so robust that the addition of a dialkylated perylene monoimide moiety at the end of the pillar does not change the packing behavior of the MSW on the substrate. We succeeded in anchoring the perylene imide in such a shape‐persistent manner that it does neither approach surface nor MSW backbone in both experiment and simulation. The shape‐persistent linker in **3** mediates a robust decoupling of the dye unit from the substrate despite its extended length.

These results rose the question of the behavior of **1**, **2**, and **3** in more complex environments, such as in binary mixtures with **4**. The components might form separate phases, or 2D cocrystals, such as observed for **4** and its corresponding cyclotrimer.^[8b]^ When we apply a solution containing **1** and **4** (*c*_1_=*c*_4_=1×10^−7^ m) in TCB to HOPG (at r.t.), a 2D crystalline domain of >75^2^ nm^2^ of a porous chiral Kagomé pattern is formed (Figure 5 a/d/g/h). To this, a unit cell with *a*=*b*=(12.0±0.3) nm, *γ*(*a*,*b*)=(60±2)° containing two molecules of **1** (with backbone orientations *c*_A_ and *c*_C_ and *γ*(*c*_A_,*c*_C_)=60°, *c*_C_ ∥ *d*_1_, cf. Figure 5 h) and one molecule of **4** is indexed, and which is oriented relative to the HOPG main axis *d*_1_ with *γ*(*a*,*d*_1_)=(25±2)°. Like for the pure compounds (cf. Figure 3 a/h/k/l), the side chains align along the latter and interdigitate intermolecularly, so that each molecule of **1** (or **4**) interacts with three (six) molecules of **4** (**1**) via ABAB side chain interdigitation motifs (cf. Figure 5 g). All innermost side chains relative to the intermolecular nanopores are either oriented in counterclockwise ((+), Figure 5 g) or clockwise (−), fashion. Again, the propargyl alcohol functions are clearly visible. Moreover, the smaller intermolecular (and hexagonal as well as pentagonal intramolecular) nanopores in the cocrystal (cf. Figure 5 a/d/g) lead to a confinement of motion of 21 (and 19 as well as 3×2) intercalated TCB solvent molecules and appear as medium‐bright punctate features.^[23]^ In contrast, the nonspecific intercalation of **1** (possible in the honeycomb pattern of pure **1**, cf. Figure 3 a) is effectively prevented due to a ratio of adsorbate (here: **1**) to pore dimensions of >1, so that (in contrast to the nanopatterns of pure **1** to **3**) the distances between two adjacent pillar units in the Kagomé cocrystals are both better defined and increased by 15 % to *a*/√3=(6.9±0.1) nm.

**Figure 5:**
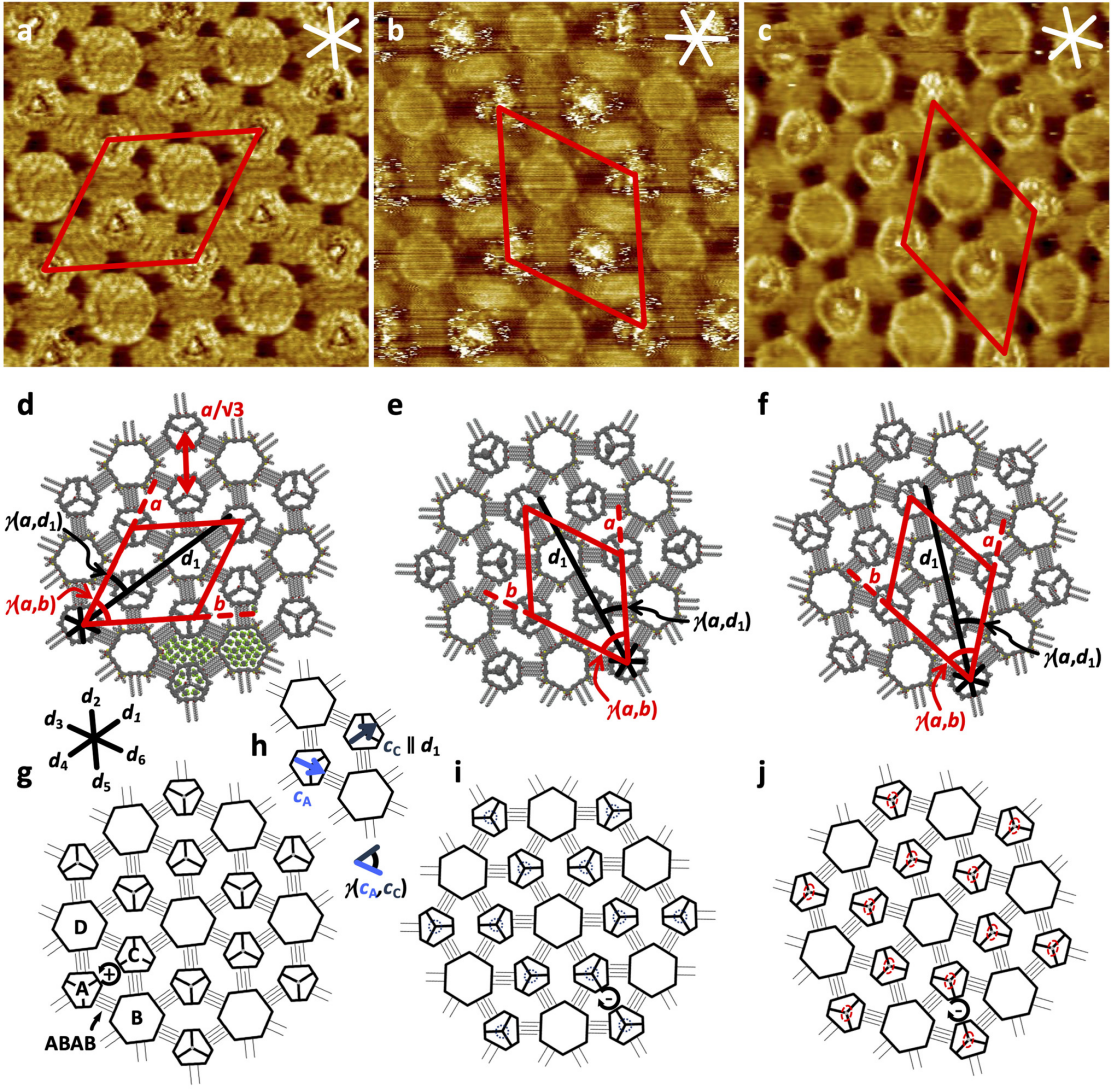
STM images, (supra‐) molecular, and schematic models of self‐assembled monolayers of mixtures of **1**–**3** and **4** at the solid–liquid interface of the respective compounds in TCB and HOPG. All monolayers were prepared at r.t. a), d), g) **1** and **4** (*V*_S_=−1.0 V, *I*_t_=25 pA, *c*_1_=1×10^−7^ m, *c*_4_=1×10^−7^ m, *c*_1_/*c*_4_=1/1; unit cell: *a*=*b*=(12.0±0.3) nm, *γ*(*a*,*b*)=(60±2)°, additional packing parameters: *γ*(*a*,*d*_1_)=(25±2)°; *a*/√*3*=(6.9±0.1) nm, ABAB indicates the side chain interdigitation scheme for molecules A and B, backbone orientations *c*_A_ and *c*_C_ (defined as bisectors) adopt relative orientations *γ*(*c*_A_,*c*_C_)=60° mediated by hexagon B; *c*_C_ ∥ *d*_1_); b), e), h) **2** and **4** (*V*_S_=−1.0 V, *I*_t_=42 pA, *c*_2_=1×10^−7^ m, *c*_4_=1×10^−7^ m, *c*_2_/*c*_4_=1/1; unit cell: *a*=*b*=(12.0±0.3) nm, *γ*(*a*,*b*)=(60±2)°, additional packing parameters: *γ*(*a*,*d*_1_)=(25±2)°); c), f), i) **1** and **4** (c: *V*_S_=−0.8 V, *I*_t_=30 pA, *c*_3_=3×10^−7^ m, *c*_4_=1×10^−7^ m, *c*_3_/*c*_4_=3/1; unit cell: *a*=*b*=(12.0±0.3) nm, *γ*(*a*,*b*)=(60±2)°, additional packing parameters: *γ*(*a*,*d*_1_)=(25±2)°). Circular arrows in g), h), i) indicate counterclockwise (+) and clockwise (−) orientation of the OC_16_H_33_ side chains that border the nanopore.^[15]^ All STM images: 30.0×30.0 nm^2^. The red and white (black) lines indicate the unit cells (*a*, *b*) and HOPG main axis directions (*d_n_*), respectively.

Similarly, **2** and **4** (*c*_2_=*c*_4_=1×10^−7^ m) as well as **3** and **4** (*c*_3_=3×10^−7^ m, *c*_4_=1×10^−7^ m, *c*_3_/*c*_4_=3/1) also form cocrystalline domains in the range of 70^2^ nm^2^ (see SI) at the solid–liquid interface of HOPG and a solution of both compounds in TCB. To the binary mixture of **2** and **4** (Figure 5 b/e/i), a unit cell with identical lattice parameters *a*=*b*=(12.0±0.3) nm, *γ*(*a*,*b*)=(60±2)° and *γ*(*a*,*d*_1_)=(25±2)° containing two molecules of **2** and one molecule of **4** is indexed. While the backbones of the molecular hexagons appear only marginally brighter than the graphite template, 30 to 90 % of the scan lines on top of the fullerene‐substituted MSWs appear with maximum brightness. To the binary mixture of **3** and **4** (Figure 5 c/f/j), an alike unit cell (*a*=*b*=(12.0±0.3) nm, *γ*(*a*,*b*)=(60±2)° and *γ*(*a*,*d*_1_)=(25±2)°) as for the other cocrystals is indexed. The backbones of both molecules appear medium bright, but (similar to the pure packing of **3**) bright punctate contrast features are observed and attributed to the central 3D unit. Remarkably, as for pure **3**, the perylene monoimide groups do not disturb the visibility of the MSW platform and hexagon significantly despite their ≈3 nm extension and scissors cut STM tips (with low aspect ratio) used.

In summary, we have paved the way for the non‐covalent attachment of periodically arranged propargyl alcohol groups to HOPG at the solid–liquid interface. The latter are attached to pillar units which are covalently attached to the central hubs of molecular spoke wheels. These act as templates by forming 2D crystals via their alkoxy side chains that are pre‐oriented in three lateral directions, or cocrystals with molecular hexagons, then with a lattice expansion of 15 % and preventing non‐specific adsorption on interstitial sites. While the fullerene substituents, flexibly attached as butanoates, are freely movable with a radius of 2.5 nm around each stator unit without contacting each other, we showed that a more rigid attachment of a perylene monoimide substituent mediates its mechanical and also electronic decoupling. The experimental results are supported by state‐of‐the‐art GFN‐FF calculations, indicating the high theoretical predictability of the dynamics of our systems having a molecular weight of >1.6×10^4^ amu.

## Conflict of interest

The authors declare no conflict of interest.

## Supporting information

As a service to our authors and readers, this journal provides supporting information supplied by the authors. Such materials are peer reviewed and may be re‐organized for online delivery, but are not copy‐edited or typeset. Technical support issues arising from supporting information (other than missing files) should be addressed to the authors.

Supporting Information

Supporting Information

Supporting Information

Supporting Information
