# Supplementary material for: Supramolecular Nanopatterns of Molecular Spoked Wheels with Orthogonal Pillars: The Observation of a Fullerene Haze
Source: Angew Chem Int Ed Engl. 2021 Nov 23;60(52):27264–70. doi: 10.1002/anie.202111869 (PMC9298702; doi:10.1002/anie.202111869)
Supplement: Supplementary file 3 — Supporting Information [file ANIE-60-27264-s004.pdf]

Angewandte Chemie
Eine Zeitschrift der Gesellschaft Deutscher Chemiker

Supporting Information

Supramolecular Nanopatterns of Molecular Spoked Wheels with Orthogonal Pillars: The Observation of a Fullerene Haze

Georgiy Poluektov, Tristan J. Keller, Anna Jochemich, Anna Krönert, Ute Müller, Sebastian Spicher, Stefan Grimme, Stefan-S. Jester,* and Sigurd Höger**

anie_202111869_sm_miscellaneous_information.pdf
anie_202111869_sm_MD_of_2.mp4
anie_202111869_sm_MD_of_3.mp4
anie_202111869_sm_xyz.zip

Contents

**1**	**General information**	**S3**
1.1	Materials and equipment	S3
1.2	GPC experiment	S3
1.3	STM experiment	S3
1.4	Computational details	S4
**2**	**Additional STM images**	**S5**
2.1	Overview STM image of **1**	S5
2.2	Overview STM image of **2**	S6
2.3	Overview STM image of **3**	S7
2.4	Bias voltage dependence of STM images of **2**	S8
2.5	Bias voltage dependence of STM images of **3**	S10
2.6	Topgraphies of **1** and **3**	S12
2.7	Overview STM image of the binary mixture of **1** and **4**	S13
2.8	Overview STM image of the binary mixture of **2** and **4**	S14
2.9	Overview STM image of the binary mixture of **3** and **4**	S15
**3**	**Synthesis**	**S16**
3.1	Synthesis of **1**, **2**, and **3**	S16
3.2	Synthesis of **5**	S31
3.3	Synthesis of **17**	S35
3.4	Synthesis of **6a** and **6b**	S39
3.5	Synthesis of **11**	S42
3.6	Synthesis of **4**	S45
**4**	**References**	**S46**

1 General information

1.1 Materials and equipment

Reagents were purchased at reagent grade from commercial sources and used without further purification. All air-sensitive reactions were carried out using standard Schlenk techniques under argon. [(3-Cyanopropyl)diisopropylsilyl]acetylene (CPD*i*PS-acetylene) and [(3-cyanopropyl)dimethylsilyl]acetylene (CPDMS-acetylene) were synthesized according to literature procedures described in [S1]. Reaction solvents (THF, piperidine, dichloromethane, pyridine, triethylamine, toluene) were dried, distilled, and stored under argon according to standard methods; workup solvents were either used in "p.a." quality or purified by distillation (dichloromethane, cyclohexane). Prior to characterization and further processing, all solids and oils were dried at r.t. under vacuum. ^{1}H and ^{13}C NMR spectra were recorded on a Bruker Avance I 300 MHz, Bruker Avance I 400 MHz, Bruker Avance III HD 500 MHz Prodigy and Bruker Avance III HD 700 MHz Cryo (300.1, 400.1, 500.1 and 700.1 MHz for ^{1}H and 75.5, 100.6, 125.8 and 176.0 MHz for ^{13}C). Chemical shifts are given in parts per million (ppm) referenced to residual ^{1}H or ^{13}C signals in deuterated solvents. All NMR spectra were recorded at r.t. unless otherwise described. Mass spectra were measured on a Finnigan ThermoQuest MAT 95 XL (EI-MS), a Sektorfeldgerät MAT 90 (EI-MS), a Bruker Daltonics micrOTOF-Q (ESI-MS, APCI), a Thermo Fisher Scientific Orbitrap XL mass spectrometer (ESI-MS), a Bruker Daltronics autoflex TOF/TOF (MALDI-MS; matrix material: DCTB, no salts added) and an ultrafleXtreme TOF/TOF of the Bruker Daltonik company (MALDI-MS; matrix material: DCTB, no salts added). *m*/*z* peaks smaller than 10 % (compared to the basis peak) are not reported. Thin layer chromatography was conducted on silica gel coated aluminium plates (Macherey-Nagel, Alugram SIL G/UV254, 0.25 mm coating with fluorescence indicator). Silica gel Kieselgel 60 (Merck, 0.040-0.063 mm) was used as the stationary phase for column chromatography. UV/vis absorption spectra were recorded on a Perkin Elmer Lambda 18 and fluorescence emission spectra on a Perkin Elmer LS-50B spectrophotometer using 10 mm quartz cuvettes. Microwave assisted reactions were performed in a CEM Discover Labmate instrument (maximal power: 300 W). Melting points were measured using an optical microscope equipped with a heating table (Leica DMLB, Leica LMW, Testo 965).

1.2 GPC experiment

Gel permeation chromatography (GPC) was performed in THF (HPLC grade, stabilized with 2.5 ppm BHT) at r.t. GPC analyses were run on an Agilent Technologies system at a flow rate of 1 mL/min using an IsoPump (G1310 A), a diode array UV detector (G1315B) and PSS columns (Polymer Standards Service, Mainz, Germany; 10^2, 10^3, 10^5 and 10^6 Å, 5 µ, 8 × 300 mm). All molecular weights were determined versus PS calibration (PS standards from PSS, Mainz, Germany).

For the preparative separation, a Shimadzu Recycling GPC system, equipped with an LC-20 AD pump, an SPD-20 A UV detector and a set of three preparative columns from PSS (either SDV 10^3 Å, 5 µ, 20 × 300 mm or SDV preparative linear S, 5 µ, 20 × 300 mm) with precolumn (SDV, 5 µ, 20 × 50 mm) was employed. The system operated at a flow rate of 5 mL/min, THF, 35 °C.

1.3 STM experiment

Scanning tunneling microscopy (STM) was performed under ambient conditions (r.t.) at the solution/solid interface, using 1,2,4-trichlorobenzene (TCB) as solvent and highly oriented pyrolytic graphite (HOPG) as substrate. In a typical experiment, 0.2 µL of a 1×10^{-7} M to 3×10^{-5} M solution of

the compound(s) of interest was dropped onto a freshly cleaved HOPG substrate at r.t. or at elevated temperature (80 °C), kept at this temperature for 10 s to 20 s, and allowed to cool to r.t. before the STM measurements were performed with the tip immersed into the solution. Bias voltages between –1.4 V and +1.1 V and tunneling current set points in the range of 9 pA to 55 pA were applied to image the supramolecular adlayers shown here. The experimental setup consists of an Agilent 5500 scanning probe microscope that is placed on a Halcyonics actively isolated microscopy workstation. It is acoustically shielded with a home-built box. Scissors cut Pt/Ir (80/20) tips were used and further modified after approach by applying short voltage pulses until the desired resolution was achieved. HOPG was obtained from TipsNano (*via* Anfatec) in ZYB-SS and DS quality. All STM images (unless otherwise noted) were calibrated by subsequent immediate acquisition of an additional image at reduced bias voltage, therefore the atomic lattice of the HOPG surface is observed which is used as a calibration grid. Data processing, also for image calibration, was performed using the SPIP 5 (Image Metrology) software package. (Supra-) molecular modelling was performed using Wavefunction Spartan ’16 and ‘18. Equilibrium geometries of the backbone structures were obtained using molecular mechanics (based on the Merck Molecular Force Field (MMFF)) and a graphene monolayer with fixed atom positions as interaction partner. Alkoxy sidechains were subsequently attached with the alkoxy-backbone angles as observed by STM.

1.4 Computational details

For the optimized input structures (see section 1.3.), geometry optimizations were performed at the GFN-FF level of theory. The resulting structures served as input for subsequent molecular dynamics (MD) simulations. MD simulations with GFN-FF were carried out for 1 ns at room temperature (298 K) employing the implicit GBSA(THF) solvation model. A time step of 2 fs at an increased hydrogen mass of 4 amu was chosen. All calculations were performed with the freely available xtb 6.4.0 program packages with default convergence criteria 10^{-7} E_h for energies and 10^{-5} $E_h \cdot Bohr^{-1}$ for gradients. All calculations were performed on Intel © Xeon E5-2660 v4 @ 2.00 GHz machines. The GFN-FF optimized structures (*xyz*-files) and the MD trajectories (mp4-files) for **2** and **3** are as separated files parts of the Supporting Information.

2 Additional STM images

2.1 Overview STM image of **1**

Self-assembled monolayers (SAMs) of **1** at the solid/liquid interface of HOPG and a solution of **1** in 1,2,4-trichlorobenzene (TCB) are investigated by STM (Figure 3a/h/k/l, Main Text, and Figure S1). At a concentration of $c = 5 \times 10^{-7}$ M, a chiral honeycomb pattern with domain sizes > 140^2 nm^2 (Figure S1) is observed. Lattice defects include missing molecules in the packing (arrow 1 in Figure S1) and unspecifically adsorbed molecules in the hexagonal nanopores (*e.g.* arrows 2 and 3 in Figure S1). The latter move within these nanopores as a result of translational degrees of freedom and are therefore observed as diffuse contrast features.

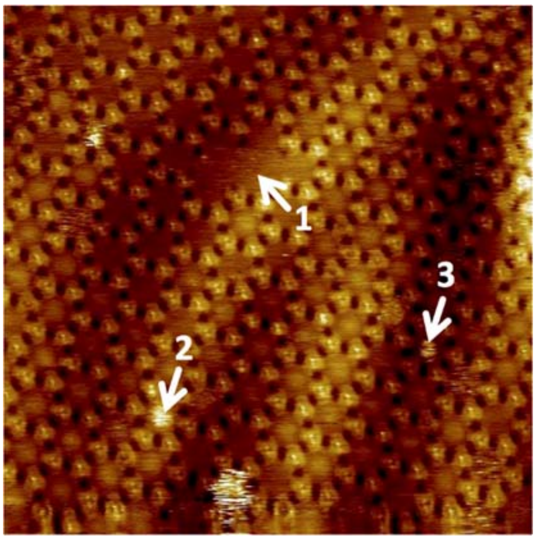

Figure S1. Overview STM image of **1** at the solid/liquid interface of HOPG and a solution of **1** using TCB as a solvent. Image parameters: 140 × 140 nm^2 (internal scanner calibration), $c = 5 \times 10^{-7}$ M, thermally annealed for 20 s to 80 °C, $V_S = -1.4$ V, $I_t = 17$ pA. Arrow 1 indicates missing molecules, arrows 2 and 3 mark examples of unspecifically adsorbed molecules of **1** located in the intermolecular nanopores.

2.2 Overview STM image of **2**

SAMs of **2** are investigated at the solid/liquid interface of HOPG and a solution of **2** in TCB by STM (Figure 3b-d/i/m, Main Text, and Figure S2). **2** is comparable to **1** in backbone size, shape, and alkoxy side chain substitution, although carrying a sterically demanding fullerene derivative substituent at its pillar unit. When a 1×10^{-7} M solution of **2** is applied to a freshly cleaved HOPG surface, a chiral honeycomb packing with domains exceeding 90^2 nm^2 lateral size (Figure S2) is observed, however with alike defects compared to **1**. These include missing molecules within the packing (*e.g.* arrow 1 in Figure S2). Moreover, one molecule in Figure S2 lacks a fullerene substituent (arrow 2). This also proves the distinguishability of three-dimensional substituents at the given imaging parameters and gives a hint on the isomorphism of **1** and **2**.

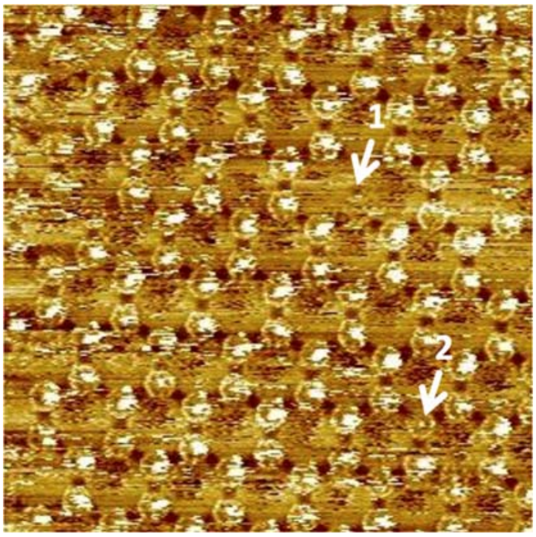

Figure S2. Overview STM image of **2** at the solid/liquid interface of HOPG and a solution of **2** using TCB as a solvent. Image parameters: 89×89 nm^2 (internal scanner calibration), $c = 1 \times 10^{-7}$ M, thermally annealed for 20 s to 80°C, $V_S = -0.80$ V, $I_t = 55$ pA. Arrow 1 indicates a packing defect (missing molecule), and arrow 2 indicates a single molecule lacking the fullerene substituent.

2.3 Overview STM image of **3**

SAMs **3** at the solid/liquid interface of HOPG and a solution of **3** in TCB are investigated by STM (Figure 3e-g/j/n in the Main Text, and Figure S3). At concentrations of $c = 1 \times 10^{-5}$ M to 5×10^{-7} M in the supernatant solution phase, a chiral honeycomb pattern is observed, and at 1×10^{-6} M, representatively, a domain size of > 72^2 nm^2 is found. As for **1** and **2**, a contrast variation in one of the intermolecular nanopores (arrow 1 in Figure S3) is observed, attributed to an unspecifically adsorbed molecule. In the center of each medium bright backbone, depending on the exact bias voltage, V_S, a small dot-shaped contrast feature is observed, attributed to the three-dimensional (3D) substituent.

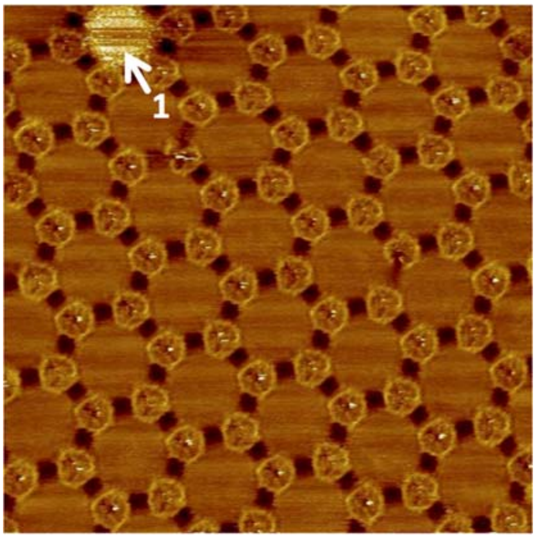

Figure S3. Overview STM image of **3** at the solid/liquid interface of HOPG and a solution of **3** using TCB as a solvent. Image parameters: 72 × 72 nm^2 (internal scanner calibration), $c = 1 \times 10^{-6}$ M, thermally annealed for 20 s to 80°C, $V_S = -0.8$ V, $I_t = 24$ pA. Arrow 1 indicates an unspecifically adsorbed molecule.

2.4 Bias voltage dependence of STM images of **2**

In the STM images shown in Figure 3b-d, Main Text, we have shown that the bias voltage does not significantly affect the visibility of the fullerene unit that is connected to the pillar unit of the MSW backbone as a butanoate. More specifically, the fullerene appears as interrupted, maximally bright scan lines superimposed to the MSW backbones, independent on whether a substrate bias voltage of V_S = –0.80 V, +1.10 V, or +0.50 V (Figures 3b-d, Main Text, respectively, and Figure S2) is applied. The HOMO and LUMO of both the MSW and the fullerene units are electronically decoupled and separated around the voltage drop, *i.e.* the connection line of the two Fermi levels of both substrate and tip, E_F^{sub} and E_F^{tip}. Moreover, the exact energetic positions of HOMOs and LUMOs obtained for isolated molecules in the gas phase vary substantially when a molecule is electronically coupled to a solid substrate, and levels are broadened. Generally, MOs located in the conduction region, which is the energetic region between both Fermi energies, lead to an increase of the tunneling current as compared to vacuum. Based on these assumptions, we propose the following energetic scenario that can explain the observed image contrast. Nominally, at a moderate negative sample bias voltage (Figure S4a and STM image in Figure 3b, Main Text, and Figure S2), the HOMO of the MSW and the fullerene should be energetically located in the conduction region, thus both molecule parts should be visible and appear bright, as they both should contribute to the tunneling current. Moreover, due to the flexible nature of the connection, the fullerene can be closer to either substrate or tip, thus the exact energetic location of its HOMO can alter, which is shown by the grey tilted bar in Figure S4a. This should, however, have no influence on its general visibility in this case. More specifically, the three-dimensional extension of **2** leads to a dominance of the fullerene once it is spatially located between the tip and the MSW and substrate at a given time, rendering as brightly appearing scan lines. However, thermal and tip-induced motion may lead its invisibility at other times, rendering as darker scan lines (of the MSW backbone, only). We accordingly refer to the random observation of bright scanlines between otherwise dark scanlines as “fullerene haze”. A similar behavior holds for a moderate positive sample bias voltage (Figure S4b, and STM image in Figure 3c, Main Text), with inverse energetic scenario. At small positive sample bias voltage (Figure S4c, and STM image in Figure 3d, Main Text), the HOMO of the MSW should energetically be located outside of the conduction region, therefore the MSW should not contribute to the overall tunneling current. However, while the actual image (Figure 3e, Main Text) is still dominated by the brightly appearing interrupted scan lines of the fullerene, the central pillar unit renders invisible as compared to Figure 3d, Main Text.

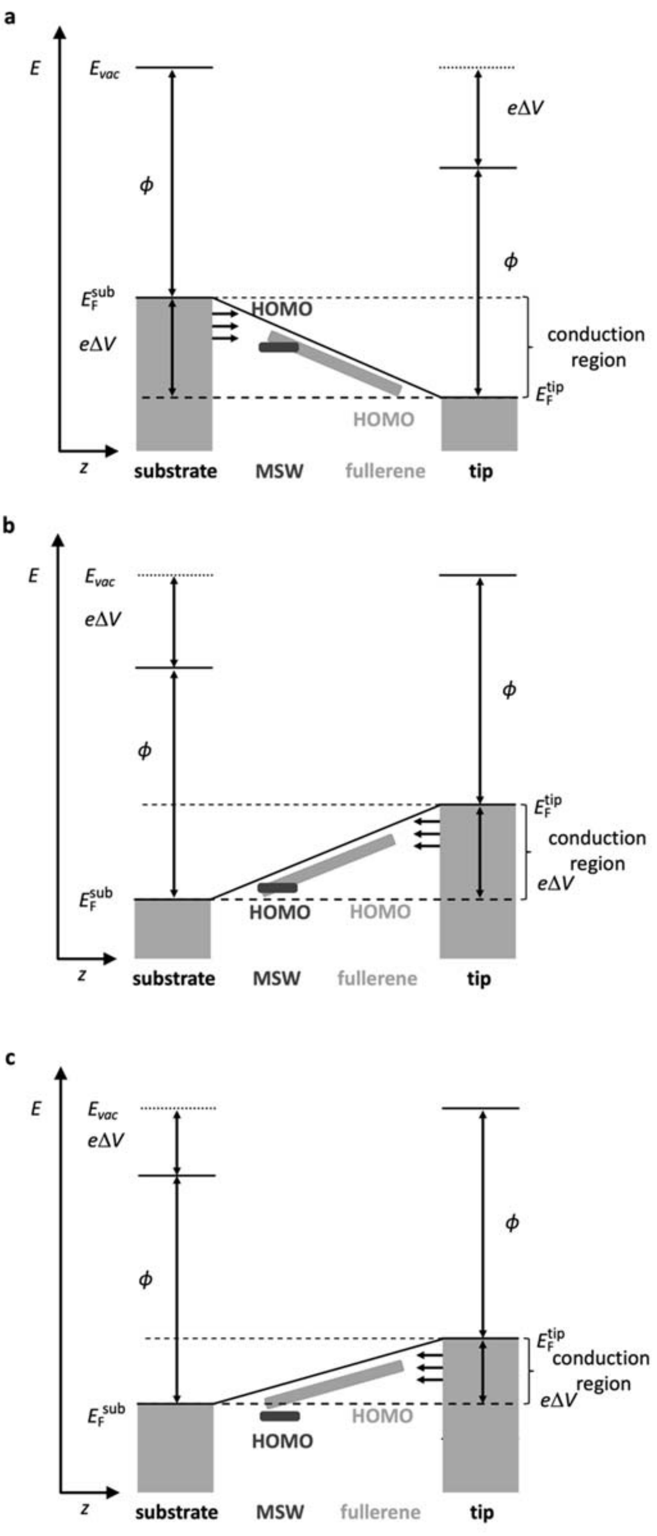

Figure S4: Proposed energetic schemes of substrate, tip, and HOMOs of the MSW and fullerene units of **2** at a) moderate negative, b) moderate positive, and c) small positive sample bias voltage. The energetic location of the HOMO of the fullerene unit varies with its spatial location, which can be closer to either substrate or tip, which is indicated by tilted grey lines.

2.5 Bias voltage dependence of STM images of **3**

In the STM images shown in Figure 3e-g (Main Text) and Figure S3, we observed a significant bias voltage dependence of the STM images of **3**. At moderate negative substrate bias voltage (of V_S = –0.61 V, cf. schematic drawing in Figure S5a and STM image in Figure 3e, Main Text), all molecule parts (*i.e.* the MSW backbone, the pillar unit carrying the perylene monoimide (PMI) unit, as well as the alkoxy side chains interdigitating intermolecularly on the HOPG substrate) are clearly visible. From this we conclude that the HOMOs of both MSW backbone and PMI/pillar units should be energetically located in the conduction region, contribute to the image contrast, and therefore might be visible by STM. The scenario should change at larger negative sample bias voltage V_S, here defined as a voltage closer to zero (of V_S = –0.50 V, cf. schematic drawing in Figure S5b and STM image in Figure 3f, Main Text): In this case, the HOMO of the 3D unit (*i.e.* PMI/pillar) may be energetically located outside (or, below) the conduction region. Consequently, the HOMO of the MSW backbone is the only orbital energetically located within the conduction region, so that only this molecule part renders visible (as seen in Figure 3e, Main Text). At small positive bias voltage (of V_S = +0.50 V, cf. schematic drawing in Figure S5c and STM image in Figure 3g, Main Text), the 3D unit dominates the image contrast, as its HOMO is energetically located in the conduction region, while the HOMO of the MSW backbone is not, and is consequently invisible (or barely visible) in the STM image.

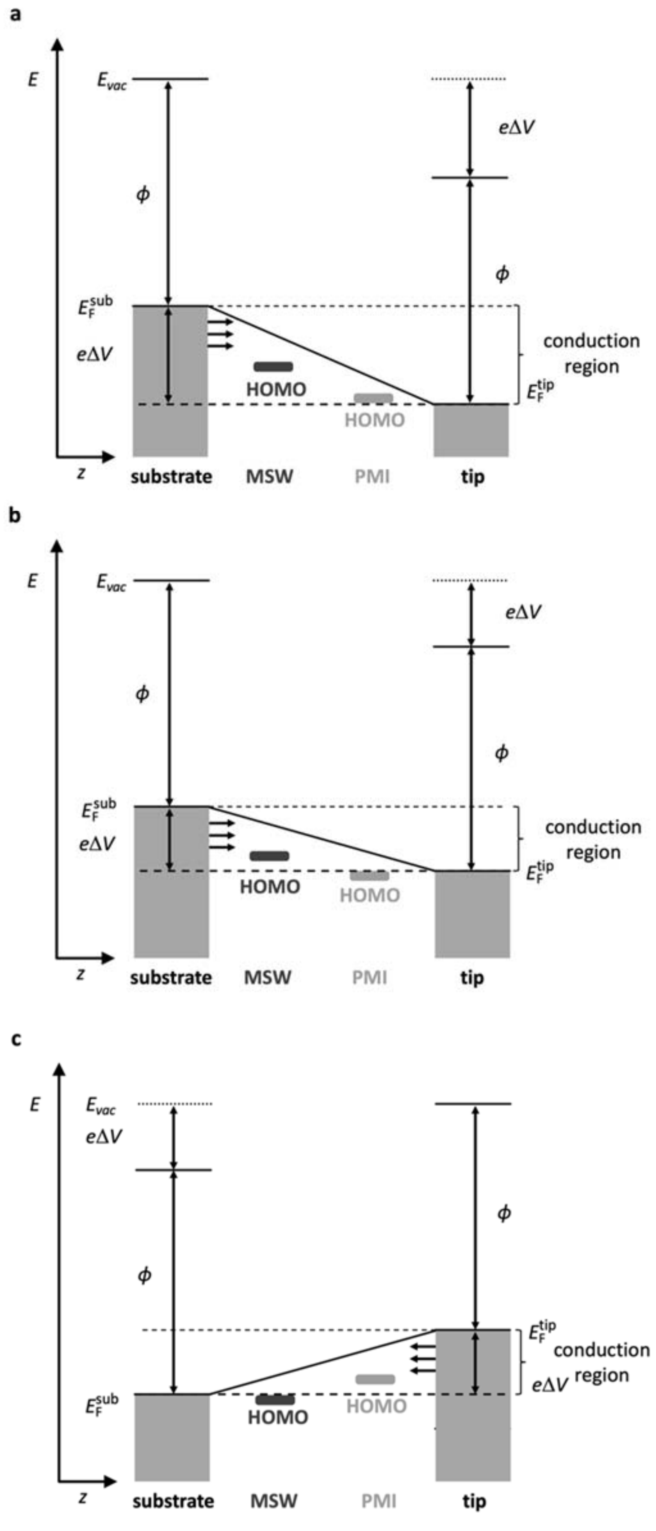

Figure S5: Energetic schemes of substrate, tip, and HOMOs of the MSW and perylene monoimide (PMI)/pillar parts of **3** at a) moderate negative, b) higher negative (*i.e.* closer to zero), and c) small positive sample bias voltage. While in a) both HOMOs of MSW backbone and PMI/pillar unit are energetically located in the conduction region, in b) only the HOMO of the MSW backbone and in c) only the HOMO of the PMI/pillar unit are energetically located in the conduction region.

2.6 Topographies of **1** and **3**

The heights of **1** and **3** measured in the STM experiments are results of the topographic heights of the adsorbed species on the HOPG surface as well as their electronic properties translating into different electrical conductivities (or tunneling resistivities), which are also bias voltage dependent. Figures S6a and b are reprints of the STM images provided in Figure 3a and e (Main Text). In Figure S6c and d we provide topography cross sections, *i.e.* apparent relative heights that are referenced to the solvent covered HOPG surface defined as zero, through the solid white lines in Figures S6a and b. The data are compared to the topographic heights obtained from the molecular models shown in Figure 2 (Main Text).

The central pillar units of **1** (Figure S6a) and **3** (Figure S6b) appear with heights of 0.14 nm and 0.24 nm, respectively. Both heights are about an order of magnitude lower than the corresponding pillar height of 1.3 nm and 2.8 nm derived from the molecular models (Figure 2a and c, Main Text), respectively. Similar behavior has already been observed for apparent heights in triazatrianguleium (TATA) derivatives.[S2] The electronic coupling of the pillar unit to the platform and/or the substrate may lead to an underestimation of the height.

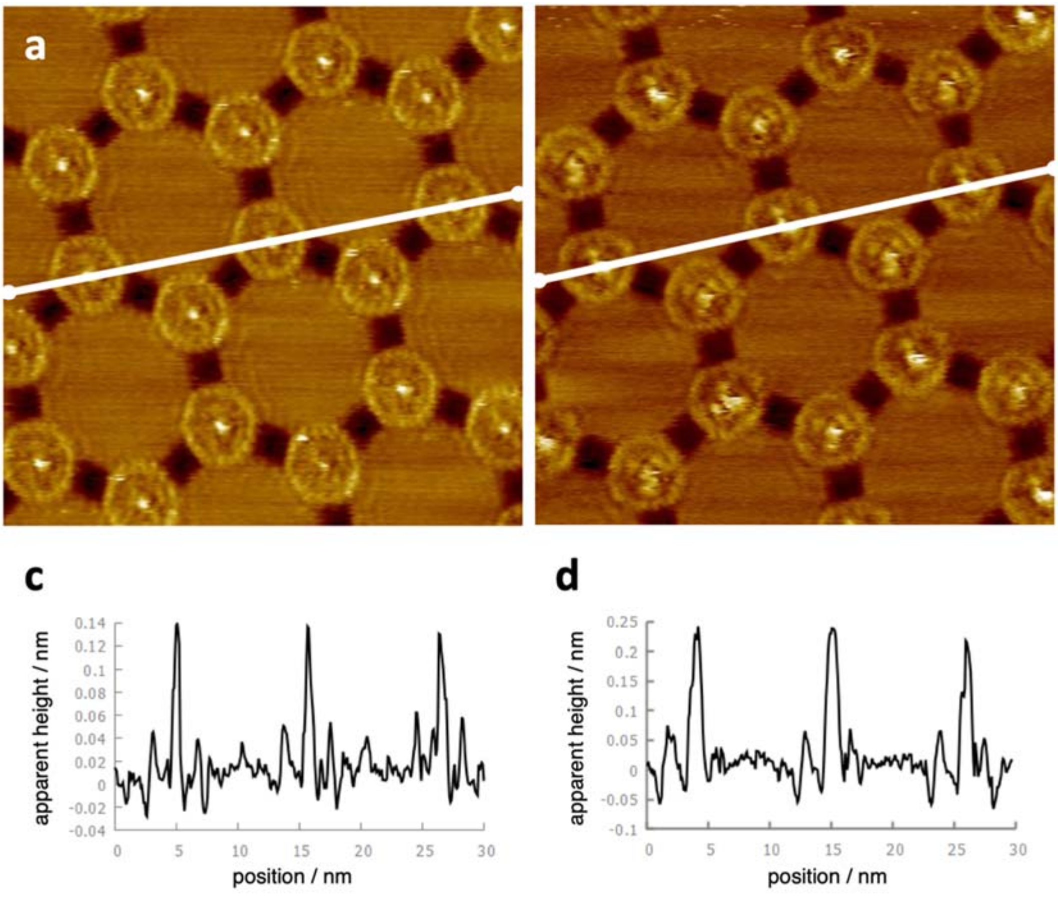

Figure S6. (a) Reprint of the STM image of **1** (Figure 3a, Main Text, image parameters: 30 × 30 nm^2, V_S = –0.70 V, I_t = 23 pA, c = 3 × 10^{-7} M); (b) reprint of the STM image of **3** (Figure 3e, Main Text, image parameters: 30 × 30 nm^2, V_S = –0.70 V, I_t = 23 pA, c = 3 × 10^{-7} M); (c) topography cross section of the line given in Figure S6a; (d) topography cross section of line given in Figure S6b.

2.7 Overview STM image of the binary mixture of **1** and **4**

When a solution of **1** and **4** (with $c_1 = c_4 = 1 \times 10^{-7}$ M) in TCB is applied to a piece of freshly cleaved HOPG at r.t., highly ordered mixed domains of a porous chiral Kagomé-pattern of > 75^2 nm^2 lateral size are observed. The backbones of each molecule (**1** and **4**) appear medium-bright in the STM image although a distinction of the species is easily feasible due to the contrast variation of maximum brightness in the center of each **1**. As described in the Main Text, each molecular hexagon **4** interacts intermolecularly *via* interdigitating side chains with six adjacent molecules of **1**, whereas each molecule of **1** interacts intermolecularly with three adjacent molecules of **4**, thus no homo-interactions between molecules of the same species are observed (except in vacancies/defects). Such defects include a molecule of **4** located on a lattice site of a molecule of **1** (cf. arrow 1, Figure S7) which, however, does not lead to a significant distortion of the 2D lattice. Moreover, a line-defect (*i.e.* homo-interaction of adjacent pillar molecules, *e.g.* arrow 2, Figure S7) is observed.

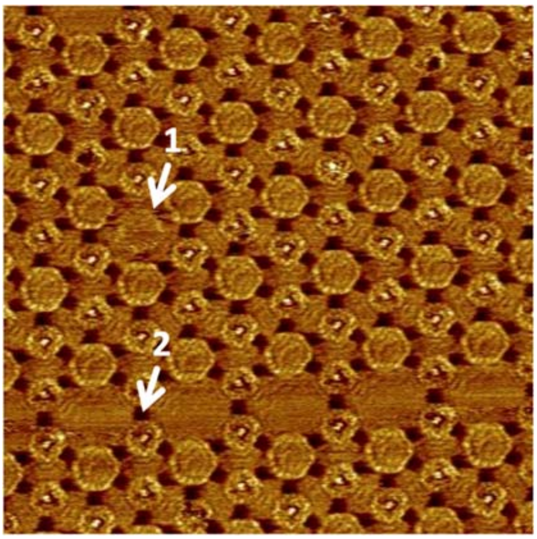

Figure S7. Overview STM image of a cocrystal of **1** and **4** at the solid/liquid interface of HOPG and a solution of **1** and **4** using TCB as a solvent. Image parameters: 75 × 75 nm^2 (internal scanner calibration), $c_1 = c_4 = 1 \times 10^{-7}$ M, adsorption at r.t. (without thermal annealing), $V_S = -1.0$ V, $I_t = 25$ pA. Arrow 1 highlights a molecular hexagon **4** located at a lattice site of a pillar molecule **1**. Arrow 2 points at a line defect (*i.e.* homo-interactions between two rows of pillar molecules without a row of **4** as mediators).

2.8 Overview STM image of the binary mixture of **2** and **4**

When a solution of **2** and **4** (with $c_2 = c_4 = 1 \times 10^{-7}$ M) in TCB is applied to a piece of freshly cleaved HOPG at r.t., a highly ordered mixed domain of a porous chiral Kagomé-pattern of ~ 70^2 nm^2 lateral size is observed (Figure S8). Depending on the exact molecule imaged, 30 % to 60 % of the fast scan lines in close proximity to the macrocycle backbone appear with maximum brightness. These brightly-appearing diffuse objects are, again, attributed to the freely jointed fullerene moiety, and allow for the feasible differentiation of **1** and **4**. The backbones of the latter appear medium-bright in the STM image and are barely distinguishable from the underlying HOPG template. Furthermore, defects within the packing such as missing molecules (*e.g.* arrow 1 in Figure S8) or a line defect (arrow 2) are observed. The observed domain is surrounded by dynamically solvent covered surface areas (regions 3 and 4).

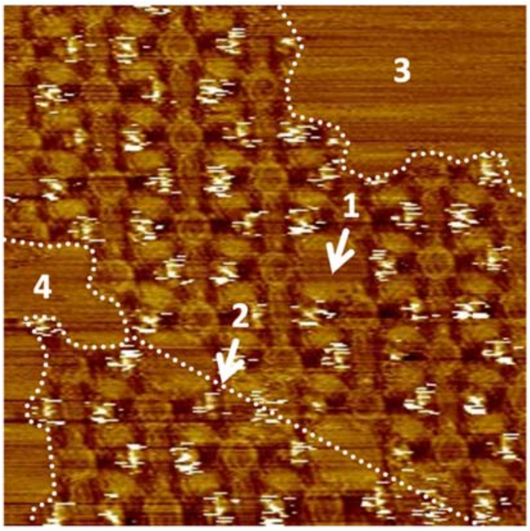

Figure S8. Overview STM image of cocrystalline domains of **2** and **4** at the solid/liquid interface of HOPG and a solution of **2** and **4** using TCB as a solvent. Image parameters: 80 × 80 nm^2 (internal scanner calibration), $c_2 = c_4 = 1 \times 10^{-7}$ M, adsorption at r.t. (without thermal annealing), $V_S = –1.0$ V, $I_t = 42$ pA. Arrows 1 marks a missing molecule, arrow 2 indicates a line defect. Arrows 4 and 5 indicate dynamically solvent covered surface areas and the island rim (also marked by white dotted lines) respectively.

2.9 Overview STM image of the binary mixture of **3** and **4**

When a solution of **3** and **4** (with c_3 = 3×10^{-7} M, $c_4 = 1 \times 10^{-7}$ M) in TCB is applied to a piece of freshly cleaved HOPG at r.t., a highly ordered mixed domain of a porous chiral Kagomé-pattern of ~ 70^2 nm^2 lateral size, surrounded by solvent molecules, is observed (Figure S9). Defects include *e.g.* a missing molecule (arrow 1), a molecule of **4** on a lattice site of **1** (arrow 2), or a deformed hexagon (arrow 3).

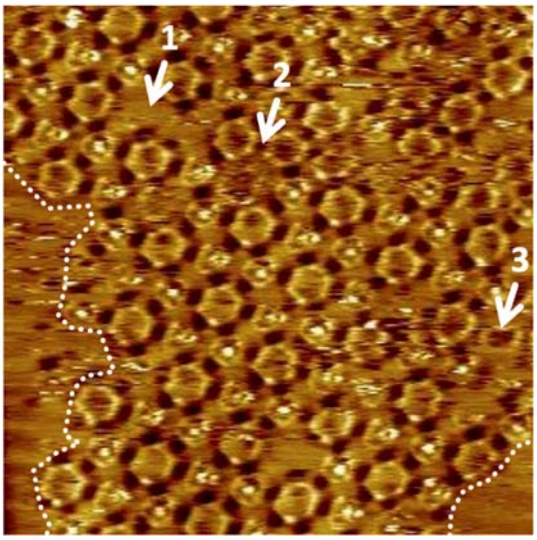

Figure S9. Overview STM image of a cocrystalline domain of **3** and **4** at the solid/liquid interface of HOPG and a solution of **3** and **4** using TCB as a solvent. Image parameters: 80×80 nm^2, $c_3 = 3 \times 10^{-7}$ M, $c_4 = 1 \times 10^{-7}$ M, adsorption at r.t. (without thermal annealing), $V_S = -1.2V$, $I_t = 30$ pA. Arrows 1, 2, and 3 highlight a missing molecule, a hexagon **4** on a lattice site of **1**, and a deformed hexagon, respectively.

3 Synthesis

3.1 Synthesis of **1**, **2**, and **3**

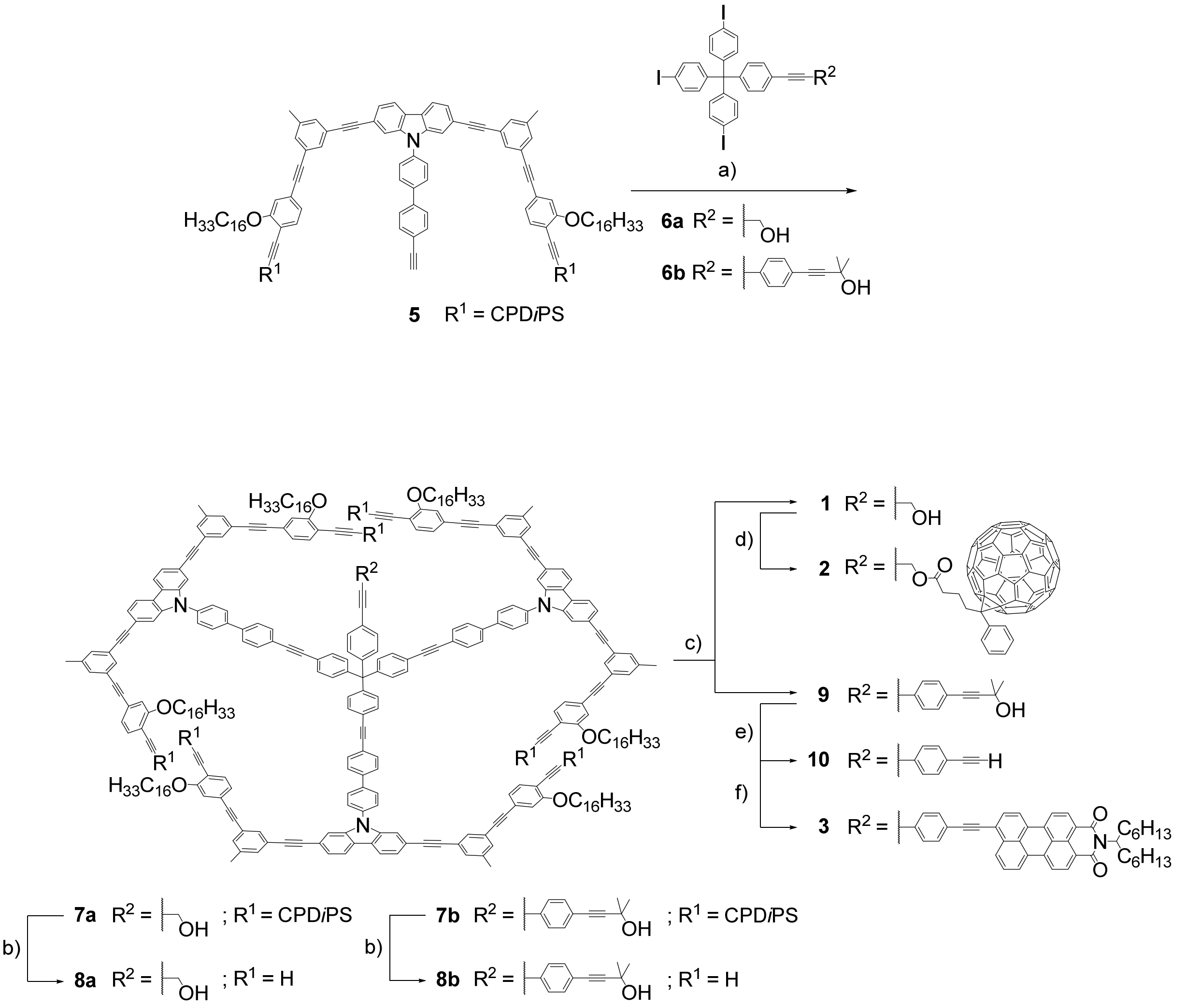

Scheme S1. a) $Pd(PPh_3)_4$, CuI, THF, piperidine, 50 °C, 18 h, 78 % (**7a**); 20 h, 89 % (**7b**); b) TBAF, THF, DCM, r.t., 1 h, 63 % (**8a**); 3 h, 57 % (**8b**); c) $PdCl_2(PPh_3)_2$, CuI, I_2, THF, $HN(i\text{-}Pr)_2$, 50 °C, 68 h, 64 % (**1**); 60 h, 32 % (**9**); d) DMAP, DCC, DCM, r.t., 17 h, 40 %; e) Bu_4NOH, MeOH, toluene, 75 °C, 2 h, 62 %; f) **11**, $Pd(PPh_3)_4$, CuI, THF, piperidine, r.t, 18 h, 30 %.

7a

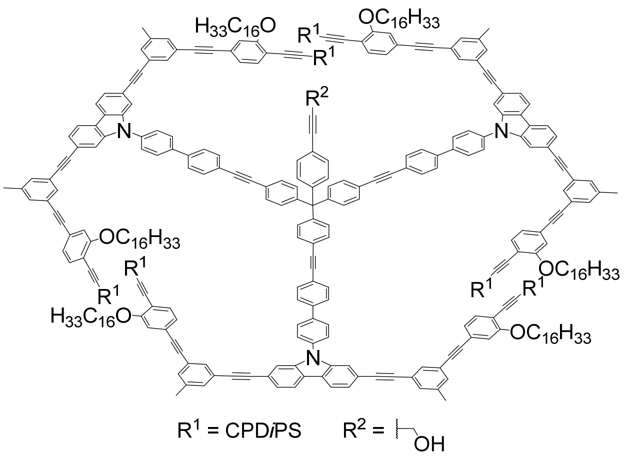

5 (79 mg, 0.10 mmol), **6a** (700 mg, 0.42 mmol), $Pd(PPh_3)_4$ (35 mg, 30 µmol) and CuI (8 mg, 40 µmol) were suspended in THF (40 mL) and piperidine (20 mL). The suspension was stirred at 50 °C for 20 h. After cooling to r.t., the reaction was diluted with water and DCM. The organic phase was separated, washed three times with HCl (1M), once with brine, and dried over $MgSO_4$. Removal of the solvent under reduced pressure and purification by column chromatography (Cy:DCM = 1:3, R_f = 0.4) yielded **7a** (420 mg, 78 µmol, 78 %) as a yellow solid.

^{1}H NMR (500 MHz, CD_2Cl_2, r.t.) δ [ppm] = 8.18 – 8.13 (m, 6H), 7.93 (d, *J* = 8.5 Hz, 6H), 7.75 (d, *J* = 8.5 Hz, 6H), 7.72 – 7.68 (m, 12H), 7.67 – 7.64 (m, 6H), 7.55 – 7.49 (m, 18H), 7.41 (d, *J* = 8.7 Hz, 2H), 7.39 (d, *J* = 7.9 Hz, 6H), 7.38 – 7.35 (m, 6H), 7.35 – 7.33 (m, 6H), 7.30 – 7.26 (m, 6H), 7.25 (d, *J* = 8.6 Hz, 2H), 7.06 (dd, *J* = 7.8 Hz, 1.4 Hz, 6H), 7.03 (d, *J* = 1.4 Hz, 6H), 4.51 – 4.44 (m, 2H), 4.02 (t, *J* = 6.4 Hz, 12H), 2.42 (t, *J* = 7.0 Hz, 12H), 2.36 (s, 18H), 1.92 – 1.75 (m, 24H), 1.53 – 1.45 (m, 12H), 1.41 – 1.20 (m, 144H), 1.18 – 1.02 (m, 84H), 0.92 – 0.77 (m, 30H).

^{13}C NMR (126 MHz, CD_2Cl_2, r.t.) δ [ppm] = 160.6, 146.7, 141.9, 140.5, 139.3, 136.9, 134.1, 132.8, 132.6, 132.1, 131.7, 131.6, 131.5, 129.2, 128.2, 127.7, 124.9, 124.6, 124.1, 124.0, 123.7, 123.2, 121.9, 121.4, 121.3, 121.2, 120.3, 115.0, 113.8, 113.5, 104.2, 96.1, 91.2, 90.7, 90.6, 90.0, 89.9, 89.3, 69.3, 65.5, 52.0, 32.5, 30.3, 30.2, 30.0, 29.9, 26.7, 23.3, 21.9, 21.4, 21.3, 21.2, 18.6, 18.3, 14.5, 12.6, 12.4, 12.1, 10.1.

MS (MALDI-TOF pos, DCTB): *m/z* calculated for $C_{376}H_{433}N_9O_7Si_6$: 5359.17; observed 5359.0.

7b

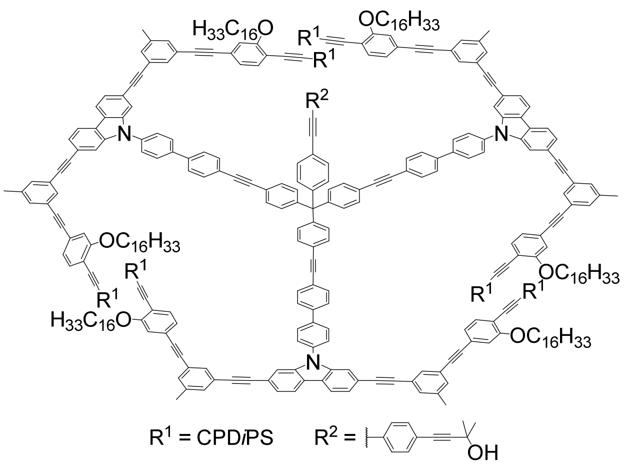

5 (851 mg, 0.511 mmol), **6b** (113 mg, 0.128 mmol), $Pd(PPh_3)_4$ (44 mg, 0.0384 mmol) and CuI (9.75 mg, 0.0512 mmol) were suspended in THF (40 mL) and piperidine (25 mL). The suspension was stirred at 50 °C for 18 h. Water and DCM were added, the organic phase was separated and washed twice with HCl (1M) and brine, and dried over Na_2SO_4. The solvent was removed under reduced pressure. After column chromatographic purification on silica gel (Cy:DCM = 1:3, R_f = 0.2), **7b** (626 mg, 0.114 mol, 89 %) was obtained as a yellow resin.

^{1}H NMR (700 MHz, CD_2Cl_2, r.t.): δ [ppm] = 8.15 (d, *J* = 8.1 Hz, 6H), 7.93 (d, *J* = 8.6 Hz, 6H), 7.76 (d, *J* = 8.5 Hz, 6H), 7.72 – 7.68 (m, 12H), 7.67 – 7.64 (m, 6H), 7.55 – 7.49 (m, 22H), 7.43 (d, *J* = 8.5 Hz, 2H), 7.38 (d, *J* = 7.8 Hz, 6H), 7.36 – 7.35 (m, 6H), 7.34 – 7.33 (m, 6H), 7.31 – 7.28 (m, 8H), 7.05 (dd, *J* = 7.8 Hz, *J* = 1.4 Hz, 6H), 7.02 (d, *J* = 1.4 Hz, 6H), 4.02 (t, *J* = 6.4 Hz, 12H), 2.41 (t, *J* = 7.0 Hz, 12H), 2.36 (s, 18H), 1.89 – 1.84 (m, 12H), 1.83 – 1.78 (m, 12H), 1.59 (s, 6H), 1.52 – 1.45 (m, 12H), 1.35 – 1.24 (m, 144H), 1.15 – 1.02 (m, 84H), 0.89 – 0.79 (m, 30H).

^{13}C NMR (176 MHz, CD_2Cl_2, r.t.) δ [ppm] = 160.6, 146.7, 141.9, 139.4, 134.1, 132.8, 132.8, 132.6, 132.1, 131.7, 131.6, 129.2, 128.2, 127.7, 124.9, 124.6, 124.0, 123.7, 123.7, 121.3, 121.2, 120.3, 115.0, 113.8, 113.5, 104.1, 96.1, 91.2, 90.7, 89.9, 89.3, 69.3, 31.9, 31.2, 29.7, 29.7, 29.7, 29.7, 29.7, 29.4, 29.4, 29.3, 26.1, 22.7, 21.4, 20.8, 20.6, 18.0, 17.8, 13.9, 11.8, 9.6.

MS (MALDI-TOF pos, DCTB): *m/z* calculated for $C_{386}H_{441}N_9O_7Si_6$: 5486.32; observed 5486.37.

8a

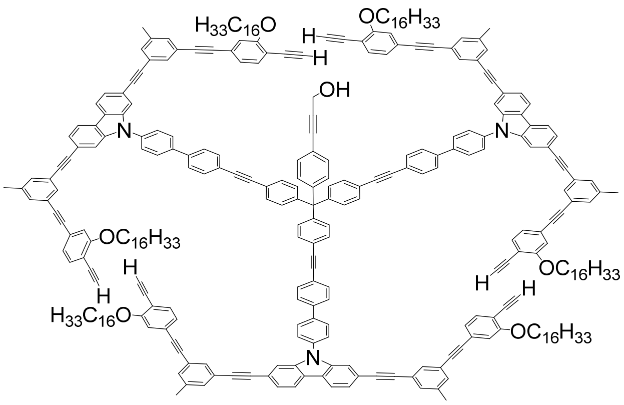

7a (280 mg, 50 µmol) was dissolved in DCM (50 mL) and TBAF (3 mL, 3 mmol, 1M in THF) was added dropwise. The solution was stirred at r.t. for 1 h and then diluted with water. The organic phase was separated, washed once with HCl (1M) and brine, and dried over $MgSO_4$. Removal of the solvent and purification by column chromatography (Cy:DCM = 1:1, R_f = 0.25) yielded **8a** (185 mg, 43 µmol, 87 %) as a yellow solid.

^{1}H NMR (400 MHz, CD_2Cl_2, r.t.) δ [ppm] = 8.18 (dd, J = 8.1 Hz, 0.7 Hz, 6H), 7.94 (d, J = 8.5 Hz, 6H), 7.79 – 7.75 (m, 6H), 7.74 – 7.67 (m, 18H), 7.58 – 7.51 (m, 18H), 7.44 (d, J = 7.8 Hz, 8H), 7.41 – 7.36 (m, 12H), 7.32 – 7.25 (m, 8H), 7.12 – 7.06 (m, 12H), 4.52 (d, J = 5.9 Hz, 2H), 4.07 (t, J = 6.6 Hz, 12H), 3.41 (s, 6H), 2.39 (s, 18H), 1.94 – 1.79 (m, 12H), 1.56 – 1.49 (m, 12H), 1.44 – 1.23 (m, 144H), 0.97 – 0.85 (m, 18H).
^{13}C NMR (101 MHz, $CDCl_3$, r.t.) δ [ppm] = 160.6, 141.9, 140.5, 139.3, 136.9, 134.4, 132.9, 132.8, 132.6, 132.1, 131.7, 131.5, 129.2, 128.2, 127.6, 125.2, 124.1, 123.7, 123.6, 121.3, 115.3, 112.6, 91.3, 90.7, 89.7, 89.3, 83.0, 69.5, 32.5, 30.3, 30.2, 29.9, 29.6, 27.5, 26.5, 23.3, 21.4, 14.5.

MS (MALDI-TOF pos, DCTB): m/z calculated for $C_{316}H_{319}N_3O_7$: 4271.04; observed 4271.0.

8b

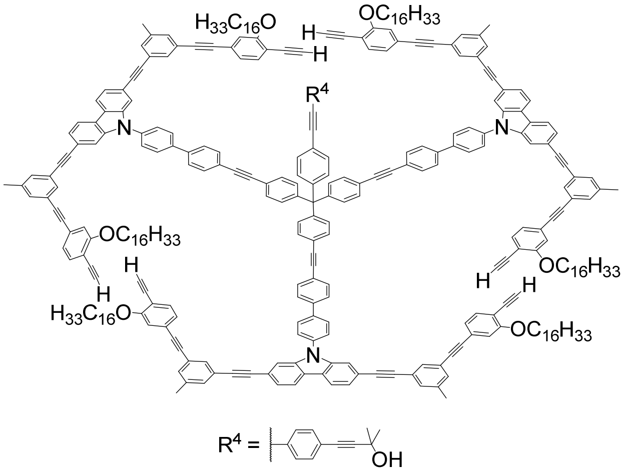

7b (851 mg, 0.155 mmol) was dissolved in DCM (70 mL) and TBAF (7.0 mL, 7 mmol, 1M in THF) was added dropwise. The solution was stirred at r.t. for 2 h and then water and DCM were added. The

organic phase was separated and washed with HCl (1M) and brine, and dried over Na_2SO_4. The solvent was removed under reduced pressure. After column chromatographic purification on silica gel (Cy:DCM = 1:2, R_f = 0.74), **8b** (390 mg, 89 μmol, 57 %) was obtained as a yellow solid.

^{1}H NMR (500 MHz, CD_2Cl_2, r.t.): δ [ppm] = 8.15 (d, *J* = 8.0 Hz, 6H), 7.92 (d, *J* = 8.6 Hz, 6H), 7.75 (d, *J* = 8.4 Hz, 6H), 7.72 – 7.67 (m, 12H), 7.66 – 7.64 (m, 6H), 7.53 (d, *J* = 8.6 Hz, 12H), 7.50 (dd, *J* = 8.0 Hz, *J* = 1.4 Hz, 10H), 7.43 (d, *J* = 8.6 Hz, 2H), 7.40 (d, *J* = 7.8 Hz, 6H), 7.38 – 7.32 (m, 12H), 7.32 – 7.27 (m, 10H), 7.06 (dd, *J* = 7.8 Hz, *J* = 1.4 Hz, 6H), 7.04 (d, *J* = 1.4 Hz, 6H), 4.04 (t, *J* = 6.6 Hz, 12H), 3.37 (s, 6H), 2.36 (s, 18H), 1.87 – 1.78 (m, 12H), 1.59 (s, 6H), 1.52 – 1.44 (m, 12H), 1.40 – 1.19 (m, 144H), 0.89 – 0.84 (m, 18H).

^{13}C NMR (126 MHz, CD_2Cl_2, r.t.) δ [ppm] = 160.6, 141.9, 139.4, 134.5, 132.9, 132.8, 132.6, 132.1, 131.7, 131.6, 129.2, 128.2, 127.7, 125.2, 124.1, 123.8, 123.7, 115.3, 112.6, 90.7, 89.7, 83.0, 80.3, 69.5, 32.5, 30.3, 30.3, 30.2, 30.2, 30.1, 29.9, 29.9, 29.6, 26.5, 23.3, 21.4, 14.5.

MS (MALDI-TOF pos, DCTB): *m/z* calculated for $C_{326}H_{327}N_3O_7$: 4398.54; observed 4398.54.

1

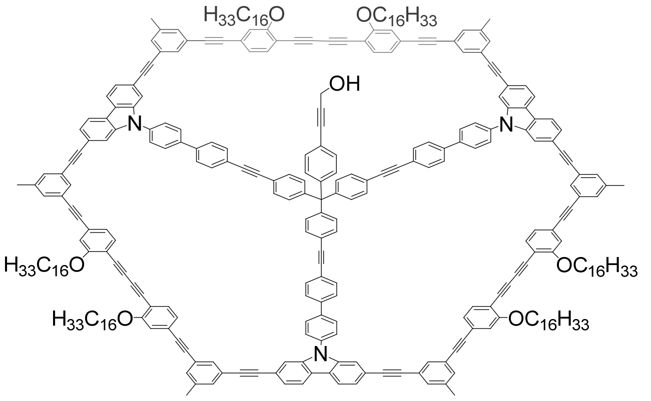

8a (50 mg, 11 mmol) in THF (30 mL) was slowly added within 48 h to a solution of $Pd(PPh_3)_2Cl_2$ (8 mg, 11 μmol), CuI (4 mg, 22 μmol), and I_2 (17 mg, 66 μmol) in THF (50 mL) and HN(*i*Pr)$_2$ (50 mL) at 50 °C. After complete addition, the reaction mixture was stirred for another 20 h and then diluted with water and DCM. The organic phase was separated and washed with aqueous HCl (1M) and brine, and dried over $MgSO_4$. Removal of the solvent under reduced pressure and pre-purification by column chromatography (DCM, R_f = 0.9) yielded the crude product which was purified by *rec*GPC and yielded **1** (30 mg, 7 μmol, 64 %) as a yellowish solid.

^{1}H NMR (400 MHz, $CDCl_3$, r.t.) *δ* [ppm] = 8.18 – 8.11 (m, 8H), 7.97 – 7.90 (m, 8H), 7.79 – 7.63 (m, 24H), 7.57 – 7.47 (m, 18H), 7.46 – 7.27 (m, 22H), 7.11 (d, *J* = 8.7 Hz, 2H), 7.07 – 7.02 (m, 12H), 4.45 (d, *J* = 6.1 Hz, 2H), 4.07 (t, *J* = 6.6 Hz, 12H), 2.39 – 2.37 (m, 18H), 1.93 – 1.82 (m, 13H), 1.45 – 1.16 (m, 152H), 0.89 – 0.79 (m, 21H).

^{13}C NMR (126 MHz, CD_2Cl_2, r.t.) δ [ppm] = 161.3, 146.4, 141.7, 140.5, 139.3, 136.9, 134.7, 132.9, 132.3, 131.7, 131.7, 129.2, 128.1, 127.6, 125.6, 124.6, 124.3, 124.1, 123.7, 123.6, 121.3, 115.3, 113.7, 112.4, 91.4, 89.8, 89.3, 79.8, 69.7, 32.5, 30.3, 30.2, 30.0, 29.6, 26.5, 23.3, 21.4, 14.5.

MS (MALDI-TOF pos, DCTB): *m/z* calculated for $C_{316}H_{313}N_3O_7$: 4264.99; observed: 4264.8.

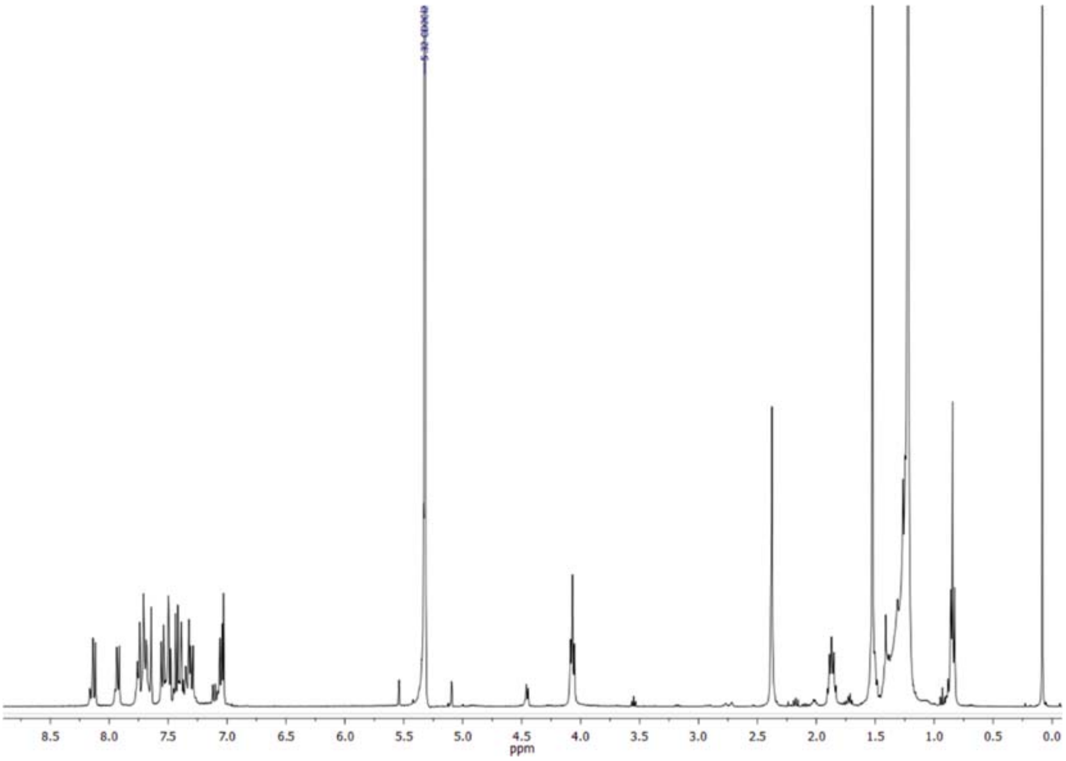

Figure S10. ^{1}H NMR spectrum of **1** (400 MHz, CD_2Cl_2, r.t.).

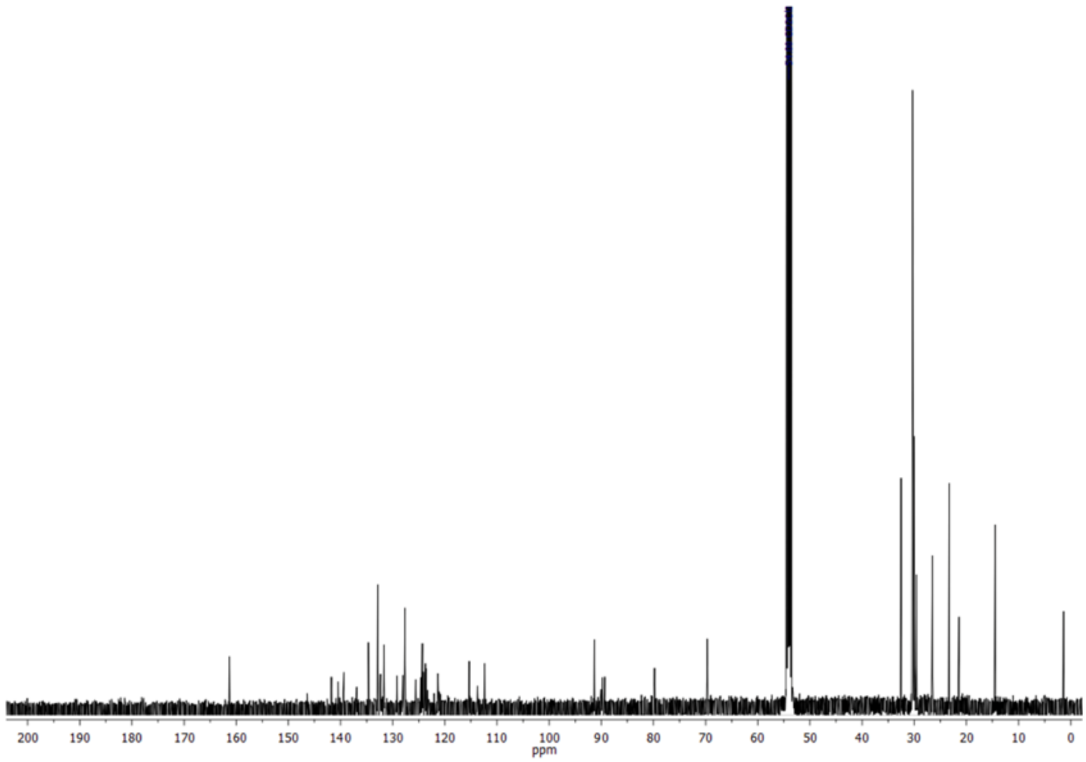

Figure S11. ^{13}C NMR spectrum of **1** (126 MHz, CD_2Cl_2 r.t.).

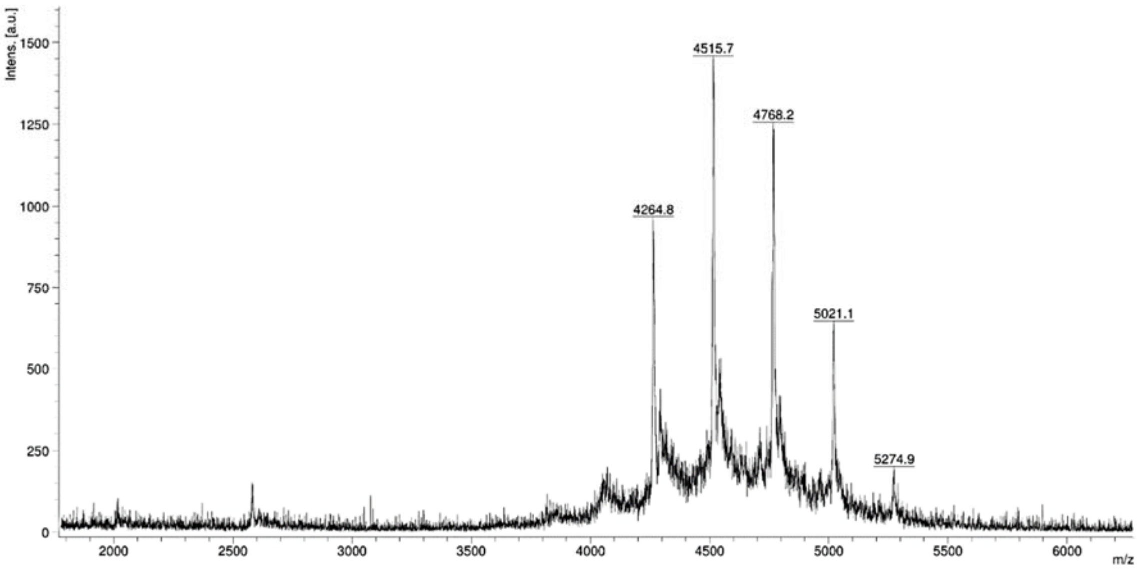

Figure S12. MALDI-TOF mass spectrum of **1** (Matrix: DCBT).

2

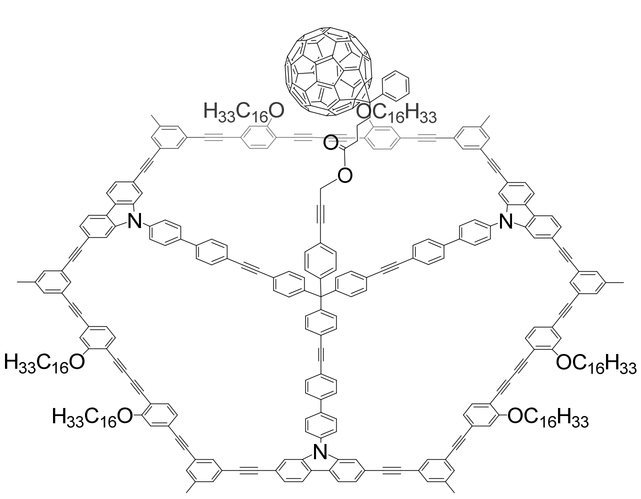

1 (20 mg, 4.7 μmol), $PC_{61}BA$ (20 mg, 23.5 μmol) and DMAP (3 mg, 23.5 μmol) were suspended in DCM (10 mL). The reaction mixture was cooled to 0 °C and DCC (5 mg, 23.5 μmol) was added. The suspension was stirred at r.t. for 17 h and then water and DCM were added. The combined organic phase was washed once with aqueous HCl (1 M) and brine, and dried over $MgSO_4$. Removal of the solvent and purification by column chromatography (Cy:DCM = 1:1, R_f = 0.62) yielded **2** (10 mg, 1.9 μmol, 40 %) as a brownish solid.

^{1}H NMR (500 MHz, CD_2Cl_2, r.t.) δ [ppm] = 8.02 (d, J = 8.1 Hz, 6H), 7.86 (dd, J = 8.1 Hz, 1.2 Hz, 6H), 7.72 – 7.65 (m, 12H), 7.59 (d, J = 8.1 Hz, 6H), 7.56 (s, 6H), 7.51 (d, J = 8.4 Hz, 6H), 7.48 – 7.35 (m, 23H), 7.35 – 7.31 (m, 8H), 7.30 – 7.24 (m, 12H), 7.08 (d, J = 8.8 Hz, 2H), 7.02 – 6.98 (m, 6H), 6.97 (d, J = 1.3 Hz, 6H), 4.87 (s, 2H), 4.04 (t, J = 6.7 Hz, 12H), 2.97 – 2.84 (m, 2H), 2.57 – 2.51 (m, 2H), 2.40 – 2.23 (m, 18H), 2.21 – 2.12 (m, 2H), 1.97 – 1.79 (m, 12H), 1.59 – 1.45 (m, 12H), 1.46 – 1.09 (m, 144H), 0.89 – 0.77 (m, 18H).

^{13}C NMR (126 MHz, CD_2Cl_2, r.t.) δ [ppm] = 172.7, 161.2, 149.5, 148.3, 147.9, 146.3, 146.2, 145.6, 145.5, 145.3, 145.2, 145.1, 144.9, 144.8, 144.5, 144.2, 143.5, 143.4, 143.3, 142.7, 142.5, 141.5, 141.4, 141.2, 140.3, 140.1, 139.2, 138.6, 137.9, 137.3, 136.8, 134.7, 132.9, 132.7, 132.3, 131.8, 131.5, 129.1, 128.9, 128.7, 127.9, 127.5, 125.5, 124.6, 124.3, 124.1, 123.7, 123.5, 123.3, 122.0, 121.2, 121.0, 115.2, 113.6, 112.3, 91.4, 90.7, 90.2, 89.9, 89.3, 80.5, 79.9, 78.2, 77.9, 77.6, 69.6, 65.5, 34.3, 34.0, 32.5, 30.3, 30.2, 30.0, 29.8, 29.6, 26.5, 23.3, 23.0, 21.4, 14.5.

MS (MALDI-TOF pos, DCTB): m/z: calculated for $C_{387}H_{323}N_3O_8$: 5143.85; observed: 5143.9.

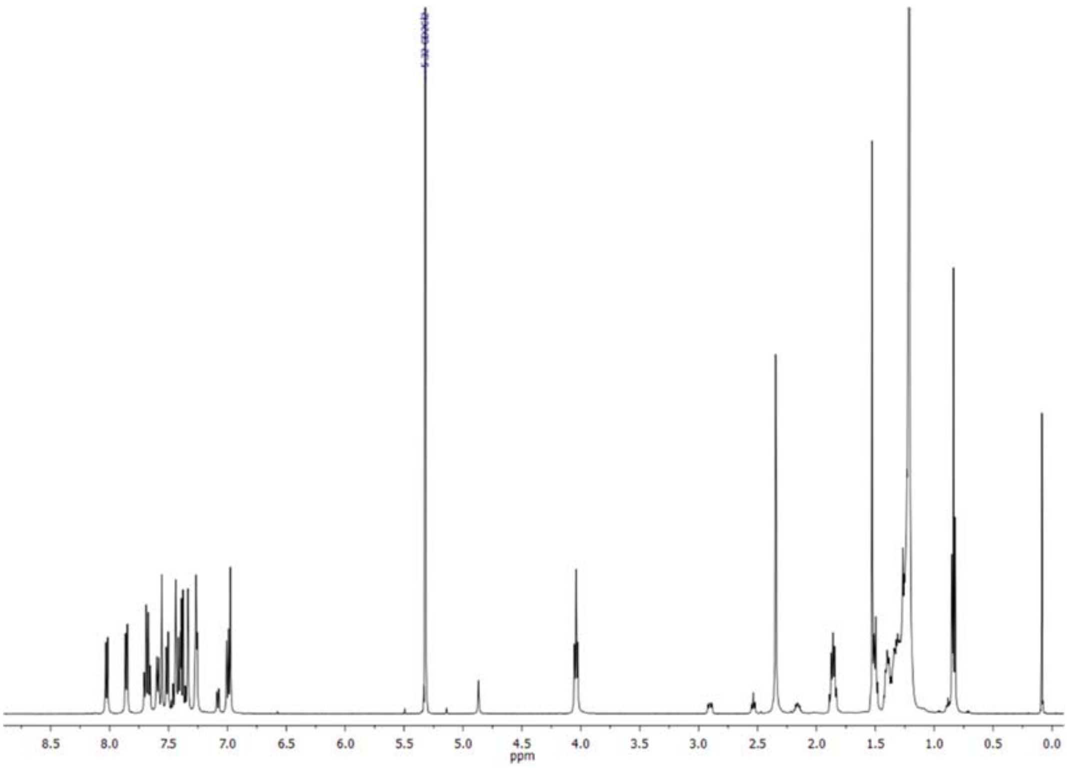

Figure S13. ^{1}H NMR spectrum of **2** (500 MHz, CD_2Cl_2 r.t.).

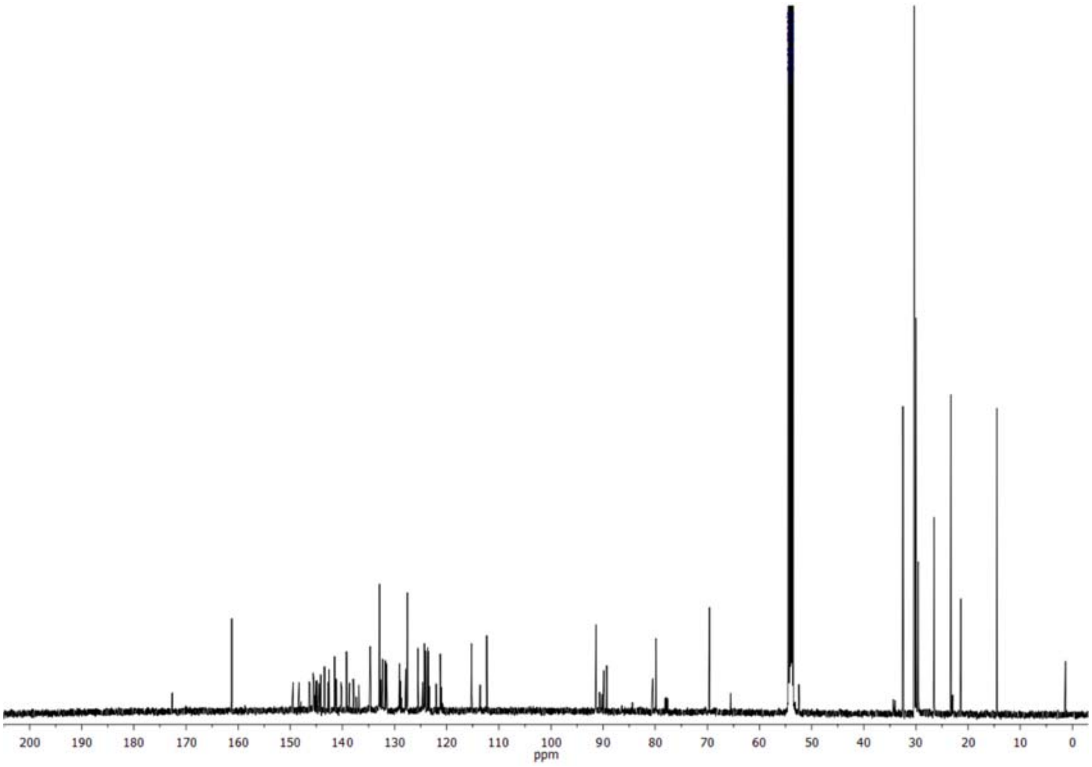

Figure S14. ^{13}C NMR spectrum of **2** (126 MHz, CD_2Cl_2 r.t.).

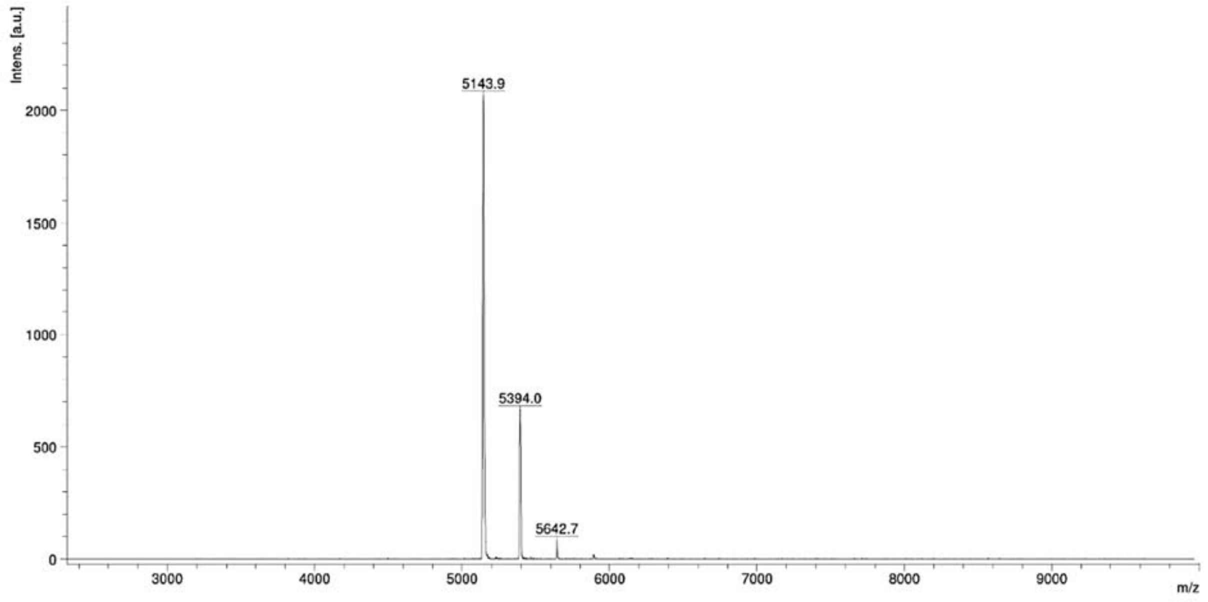

Figure S15. MALDI-TOF mass spectrum of **2** (Matrix: DCBT).

9

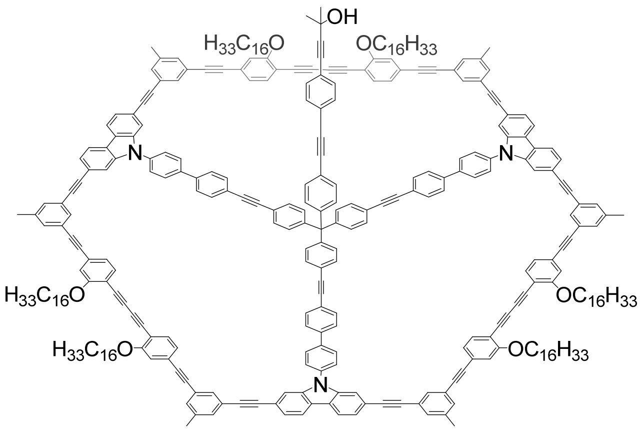

8b (50.0 mg, 11.4 μmol) in THF (30 mL) was slowly added within 60 h to a solution of $PdCl_2(PPh_3)_2$ (8.00 mg, 11.4 μmol), CuI (4.34 mg, 22.8 μmol) and I_2 (17.36 mg, 68.4 μmol) in THF (50 mL) and $HN(i\text{Pr})_2$ (50 mL) at 50 °C. Water and DCM were added, and the aqueous phase was extracted with DCM. The combined organic phase was then washed two times with HCl (1M) and once with brine, and dried over Na_2SO_4. The solvent was removed under reduced pressure and the crude product was purified by column chromatography on silica gel (DCM, $R_f = 0.6$) and subsequent purification by *rec*GPC yielded **9** (16.2 mg, 3.69 μmol, 32 %) as a yellow solid.

^{1}H NMR (700 MHz, CD_2Cl_2, r.t.): δ [ppm] = 8.03 (d, *J* = 8.0 Hz, 6H), 7.87 (d, *J* = 8.4 Hz, 6H), 7.71 (d, *J* = 8.3 Hz, 6H), 7.69 (d, *J* = 8.3 Hz, 6H), 7.60 (d, *J* = 8.4 Hz, 8H), 7.57 – 7.55 (m, 14H), 7.48 – 7.43 (m, 10H), 7.43 – 7.41 (m, 2H), 7.40 – 7.37 (m, 8H), 7.35 – 7.30 (m, 14H), 7.29 – 7.26 (m, 6H), 7.14 (d, *J* = 9.0 Hz, 2H), 7.01 (dd, *J* = 7.7 Hz, *J* = 1.4 Hz, 6H), 6.99 (d, *J* = 1.4 Hz, 6H), 4.05 (t, *J* = 6.7 Hz, 12H), 2.35 (s, 18H), 1.87 (p, *J* = 6.8 Hz, 12H), 1.57 (s, 6H), 1.55 – 1.49 (m, 24H), 1.44 – 1.37 (m, 12H), 1.36 – 1.32 (m, 12H), 1.31 – 1.28 (m, 12H), 1.26 – 1.18 (m, 94H), 0.86 – 0.82 (m, 18H).

^{13}C NMR (176 MHz, CD_2Cl_2, r.t.) δ [ppm] = 161.3, 146.3, 141.6, 139.3, 134.7, 132.9, 132.8, 132.4, 132.3, 132.1, 132.0, 131.7, 131.6, 131.6, 129.1, 127.9, 127.6, 125.5, 124.6, 124.3, 124.1, 123.7, 123.6, 123.6, 123.3, 121.3, 121.0, 115.2, 113.6, 112.3, 91.4, 90.6, 89.9, 89.3, 79.8, 69.7, 69.6, 32.5, 31.8, 30.3, 30.3, 30.3, 30.3, 30.3, 30.3, 30.3, 30.2, 23.0, 29.6, 26.5, 23.3, 21.4, 14.5, 1.3.

MS (MALDI-TOF pos, DCTB): *m/z*: calculated for $C_{326}H_{321}N_3O_7$: 4392.5; observed: 4392.5.

10

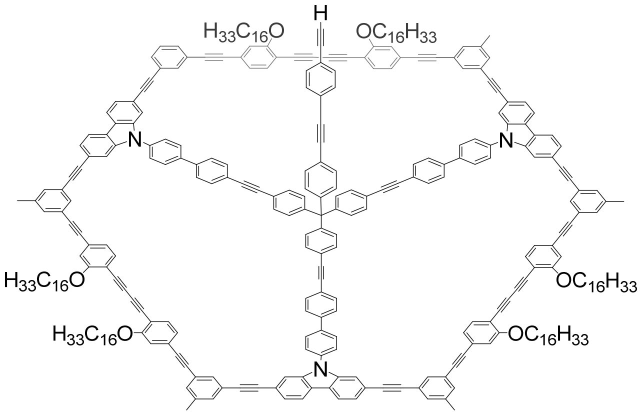

9 (16.0 mg, 3.64 μmol) was dissolved in toluene (10 mL) and heated to 75 °C. Bu_4NOH (0.1 μl, 94 μg, 0.364 μmol, 1M in MeOH) was added, and the solution was stirred at 75 °C for 5 h. Water and DCM were added, and the aqueous phase was extracted with DCM. The combined organic phase was washed twice with HCl (1M), once with brine, and dried over Na_2SO_4. The solvent was removed under reduced pressure and the crude product was purified by column chromatography on silica gel (Cy:DCM=2:1, R_f = 0.3) and yielded **10** (10 mg, 2.31 μmol, 62 %) as a yellow solid.

^{1}H NMR (300 MHz, CD_2Cl_2, r.t.): δ [ppm] = 8.03 (d, *J* = 8.0 Hz, 6H), 7.87 (d, *J* = 8.4 Hz, 6H), 7.71 (d, *J* = 8.3 Hz, 6H), 7.69 (d, *J* = 8.3 Hz, 6H), 7.60 (d, *J* = 8.4 Hz, 8H), 7.57 – 7.55 (m, 14H), 7.48 – 7.43 (m, 10H), 7.43 – 7.41 (m, 2H), 7.40 – 7.37 (m, 8H), 7.35 – 7.30 (m, 14H), 7.29 – 7.26 (m, 6H), 7.14 (d, *J* = 9.0 Hz, 2H), 7.01 (dd, *J* = 7.7 Hz, *J* = 1.4 Hz, 6H), 6.99 (d, *J* = 1.4 Hz, 6H), 4.05 (t, *J* = 6.7 Hz, 12H),3.63 (s, 1H), 2.35 (s, 18H), 1.87 (p, *J* = 6.8 Hz, 12H), 1.55 – 1.49 (m, 24H), 1.44 – 1.37 (m, 12H), 1.36 – 1.32 (m, 12H), 1.31 – 1.28 (m, 12H), 1.26 – 1.18 (m, 94H), 0.86 – 0.82 (m, 18H).

MS (MALDI-TOF pos, DCTB): *m/z*: calculated for $C_{323}H_{315}N_3O_6$: 4334.5; observed: 4334.5.

3

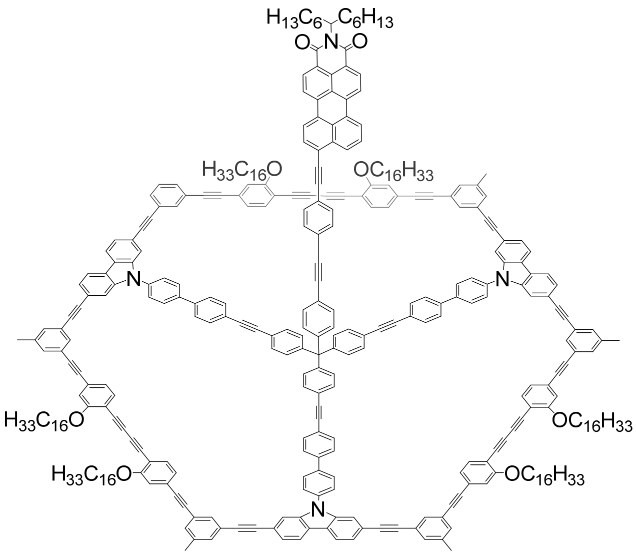

10 (10 mg, 2.31 µmol), **11** (1.45 mg, 2.30 µmol), $Pd(PPh_3)_4$ (0.13 mg, 0.115 µmol) and CuI (0.04 mg, 0.23 µmol) were suspended in THF (8 mL). Piperidine (4 mL) was added and the suspension was stirred at r.t. for 18 h. Water and DCM were added and the organic phase was separated and washed twice with HCl (1M) and brine, and dried over Na_2SO_4. The solvent was removed under reduced pressure. After column chromatographic purification on silica gel (Cy:DCM = 1:1, R_f = 0.54) and subsequent purification using *rec*GPC, **3** (3.4 mg, 0.703 µmol, 30 %) was isolated as a red solid.

^{1}H NMR (700 MHz, CD_2Cl_2, r.t.): δ [ppm] = 8.57 – 8.52 (m, 3H), 8.51 – 8.44 (m, 2H), 8.16 (d, *J* = 8.1 Hz, 6H), 7.94 (d, *J* = 8.2 Hz, 6H), 7.90 (d, *J* = 8.1 Hz, 1H), 7.77 (d, *J* = 8.3 Hz, 6H), 7.73 – 7.70 (m, 12H), 7.69 – 7.66 (m, 6H), 7.60 (d, *J* = 8.5 Hz, 2H), 7.59 – 7.55 (m, 6H), 7.53 – 7.48 (m, 14H), 7.46 – 7.42 (m, 6H), 7.42 – 7.38 (m, 6H), 7.36 – 7.32 (m, 12H), 7.19 (d, *J* = 8.6 Hz, 2H), 7.07 (dd, *J* = 7.8 Hz, 1.4 Hz, 6H), 7.04 (d, *J* = 1.4 Hz, 6H), 4.08 (t, *J* = 6.6 Hz, 12H), 2.38 (s, 18H), 2.33 (t, *J* = 7.5 Hz, 2H), 2.29 – 2.25 (m, 4H), 2.25 – 2.21 (m, 6H), 2.17 (t, *J* = 7.5 Hz, 2H), 2.04 – 1.99 (m, 4H), 1.87 (p, *J* = 6.8 Hz, 12H), 1.63 – 1.56 (m, 42H), 1.45 – 1.37 (m, 12H), 1.36 – 1.17 (m, 110H), 0.90 – 0.87 (m, 6H), 0.87 – 0.79 (m, 18H).

MS (MALDI-TOF pos, DCTB): *m/z*: calculated for $C_{358}H_{350}N_4O_8$: 4836.76; observed: 4836.8.

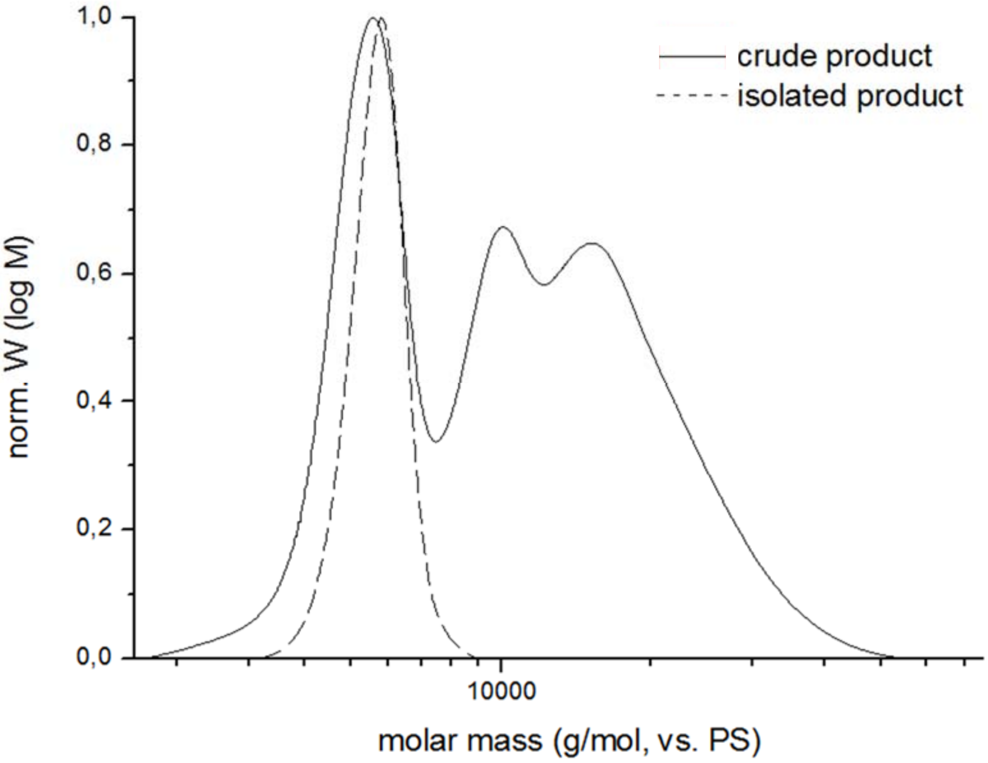

Figure S16. Molar mass distributions of GPC analyses of **3**.

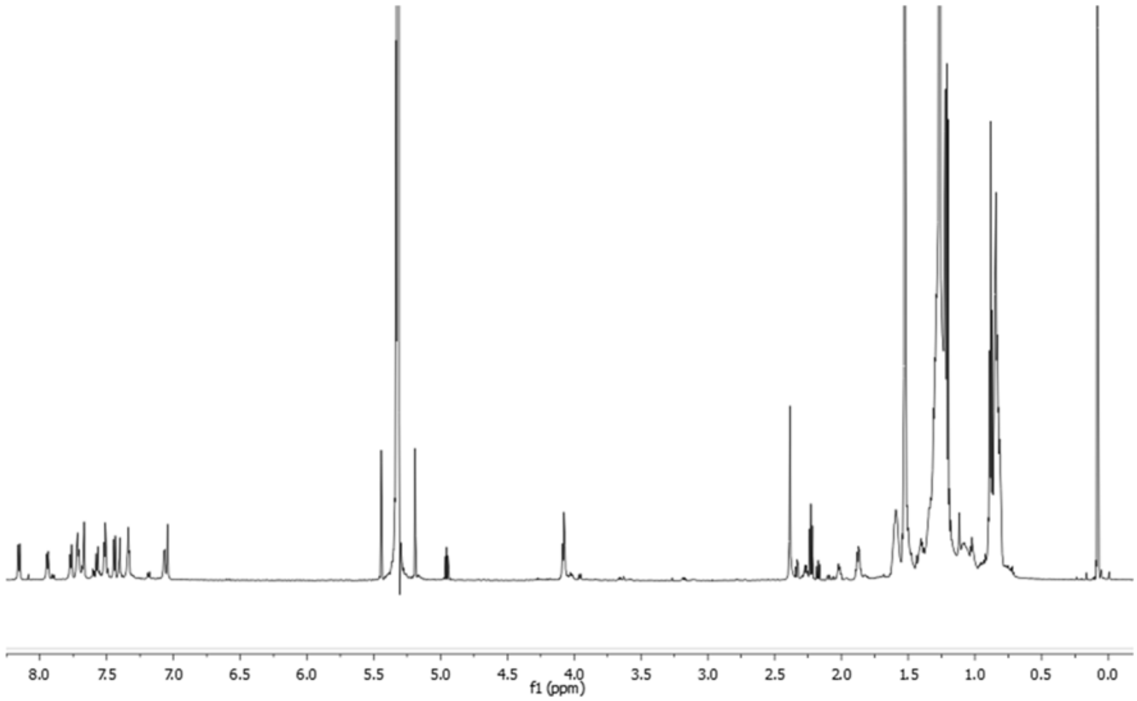

Figure S17. ^{1}H NMR spectrum of **3** (700 MHz, CD_2Cl_2 r.t.).

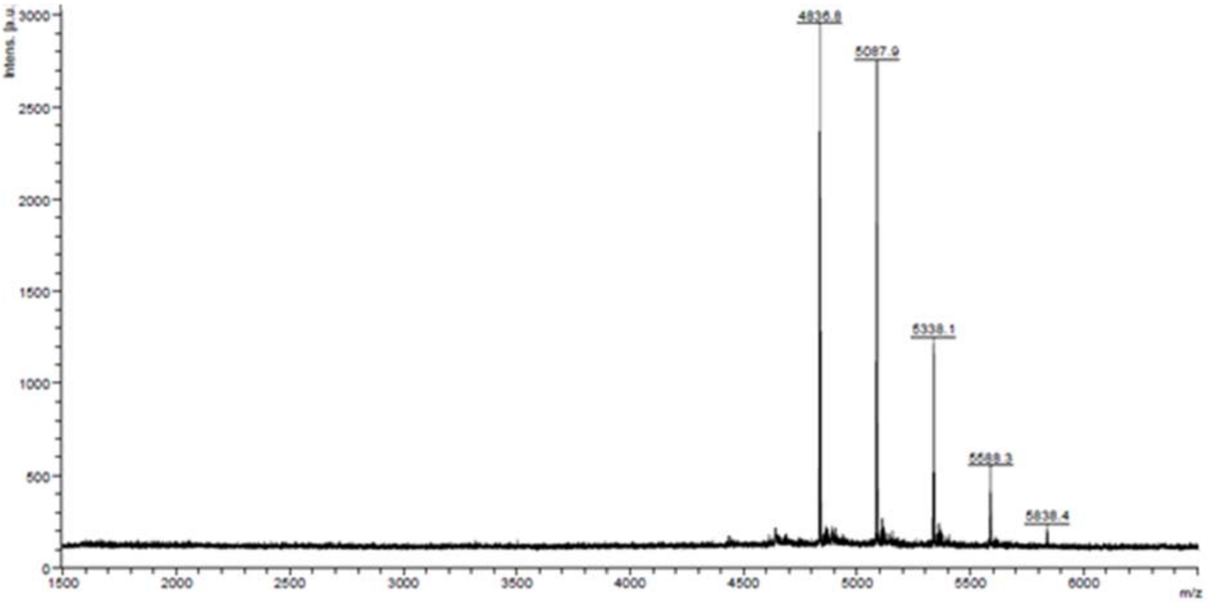

Figure S18. MALDI-TOF mass spectrum of **3** (Matrix: DCBT).

9. UV/vis absorption spectra (maxima: 350 nm; 530 nm) and fluorescence spectra (absorption maximum at 350 nm: emission maxima at 413 nm; 436 nm; 561 nm; absorption maximum at 530 nm: emission maximum at 561 nm) of **3** in THF at room temperature.

3.2 Synthesis of **5**

Coupling of 2,7-dibromocarbazole with **12** and subsequent reduction of the NO_2-group followed by a Sandmeyer-like reaction yielded **15** which was coupled with CPDMS acetylene *via* Sonogashira reaction leading to the "T"-shaped building block **16**. Twofold Sonogashira reaction with acetylene **17** followed by deprotection of the CPDMS-group yielded after deprotection of the CPDMS group the M-shaped building block **5**.

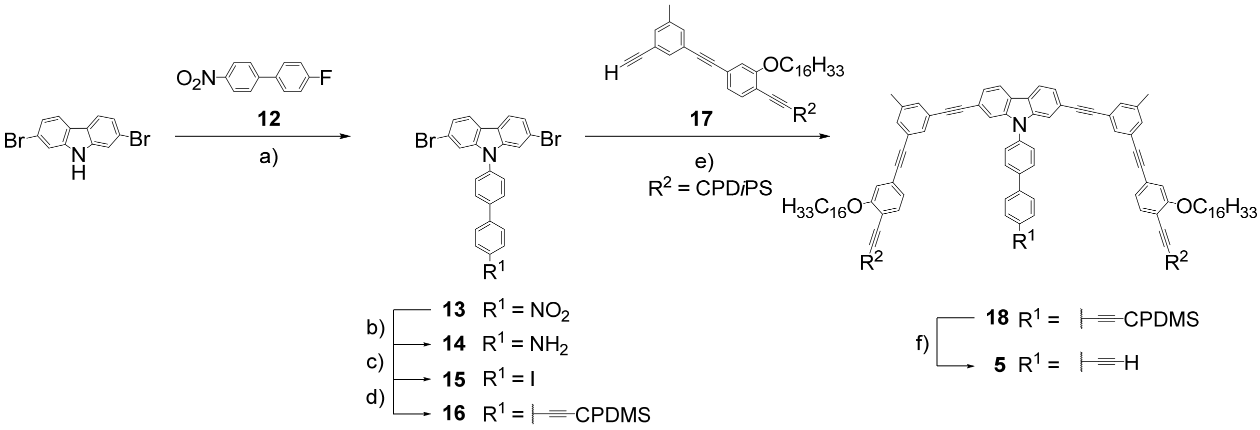

Scheme S2. a) K_2CO_3, DMF, 150 C, 22 h, 76 %; b) $SnCl_2{\cdot}H_2O$, EtOH, 80 °C, 95 h, 65 %; c) $NaNO_2$, KI, HCl, MeCN, H_2O, reflux, 22 h, 73 %; d) CPDMS acetylene, $PdCl_2(PPh_3)_2$, CuI, THF, NEt_3, rt, 2 h, 74 %; e) $Pd(PPh_3)_4$, CuI, THF, piperidine, 80 °C, 21 h, 71 %; f)) K_2CO_3, MeOH, DCM, r.t., 2 h, 63 %.

12

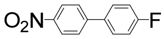

4-Fluorobenzeneboronic acid (2.8 g, 20 mmol), 4-iodonitrobenzene (5 g, 20 mmol) and K_2CO_3 (8.3 g, 60 mmol) were suspended in toluene (80 mL) and ethanol (50 mL). The suspension was purged with argon for 2 h. $Pd(PPh_3)_4$ (1.15 g, 1,00 mmol) was added, and the suspension was stirred at r.t. for 14 h. Water and DCM were added, the organic phase was separated, washed with brine, and dried over $MgSO_4$. The solvent was removed under reduced pressure, and the crude product was purified by column chromatography (Cy:DCM = 1:1, R_f = 0.46). The obtained product was dissolved in ethanol and precipitated by adding water, filtered off, and dried under vacuum. **12** (3.89 g, 17.9 mmol, 90 %) was obtained as a colorless solid.

^{1}H NMR (400 MHz, $CDCl_3$, r.t.) δ [ppm] = 8.34 - 8.25 (m, 2H), 7.73 - 7.66 (m, 2H), 7.63 - 7.56 (m, 2H), 7.23 - 7.15 (m, 2H).

^{13}C NMR (101 MHz, $CDCl_3$, r.t.) δ [ppm] = 164.7, 162.2, 146.7, 135.0, 135.0, 129.3, 129.2, 127.8, 127.8, 124.3, 116.4, 116.2.

MS (MALDI-TOF pos, DCTB): *m/z* calculated for $C_{12}H_6FNO_2$: 217.05; observed 217.0.

13

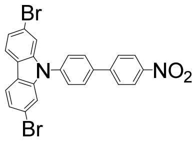

2,7-dibromo-9*H*-carbazole (1.80 g, 5.5 mmol), **4** (4.8 g, 21.1 mmol) and K_2CO_3 (3.8 g, 27.5 mmol) were suspended in DMF (125 mL), and the suspension was stirred at 150 °C for 22 h. After cooling, a solid precipitated and was filtered off. The product was recrystallized from toluene and yielded **13** (2.21 g, 4.23 mmol, 76 %) as a yellow solid.

^{1}H NMR (400 MHz, $CDCl_3$, r.t.) δ [ppm] = 8.43 - 8.35 (m, 2H), 8.00 - 7.94 (m, 2H), 7.92 - 7.83 (m, 4H), 7.69 - 7.63 (m, 2H), 7.58 - 7.54 (m, 2H), 7.47 - 7.41 (m, 2H).

^{13}C NMR (101 MHz, $CDCl_3$, r.t.) δ [ppm] = 147.6, 146.4, 141.8, 138.9, 137.3, 129.4, 128.1, 127.8, 124.5, 124.1, 122.1, 121.8, 120.3, 113.2.

MS (EI, 70 eV): *m/z* calculated for $C_{24}H_{14}Br_2N_2O_2$: 519.94; observed 522.0.

14

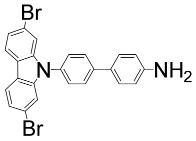

13 (1.44 g, 2.75 mmol) and $SnCl_2{\cdot}H_2O$ (3.1 g, 13.75 mmol) were suspended in ethanol (80 mL) and the suspension was purged with argon for 2 h. The suspension was was heatet to 80 °C and stirred for 95 h. After cooling to r.t., the solvent was removed under reduced pressure. DCM and NaOH solution (pH ≈ 10) were added to the resulting solid. The organic phase was separated and the aqueous phase was extracted once with DCM. The combined organic phase was dried over $MgSO_4$ and the solvent removed under reduced pressure. The product was purified *via* column chromatography (DCM, R_f = 0.54) yielding **14** (0.87 g, 1.77 mmol, 65 %) as a yellow solid.

^{1}H NMR (400 MHz, $CDCl_3$, r.t.) δ [ppm] = 8.01 – 7.96 (m, 2H), 7.85 – 7.83 (m, 2H), 7.71 – 7.65 (m, 2H), 7.63 – 7.59 (m, 2H), 7.58 – 7.55 (m, 2H), 7.47 – 7.41 (m, 2H), 7.27 (d, J = 8.6 Hz, 2H).

MS (EI, 70 eV): *m/z* calculated for $C_{24}H_{16}Br_2N_2$: 489.97; observed 489.9.

15

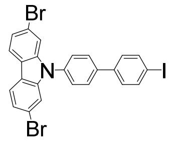

14 (0.87 g, 1.77 mmol) was suspended in MeCN (40 mL) and water (40 mL) and cooled to 0 °C. Conc. aqueous HCl (3 mL) was added dropwise. $NaNO_2$ (244 mg, 3.54 mmol) was dissolved in water (40 mL) and added dropwise to the suspension at 0 °C. The orange suspension was stirred at r.t. for 1 h. KI (1.47 g, 8.85 mmol) was dissolved in water (50 mL) was added, and the mixture was stirred under reflux for 22 h. After cooling to r.t., the reaction was diluted by adding a sodium bisulfite solution. After decolorization, DCM was added, the organic phase was separated, and the aqueous phase was extracted once with DCM. The combined organic phase was once washed with brine, dried over Na_2SO_4 and the solvent was removed under reduced pressure. Purification by column chromatography (Cy/DCM = 5:1, R_f = 0.77) yielded **15** (0.78 g, 1.29 mmol, 73 %) as a white solid.

^{1}H NMR (500 MHz, $CDCl_3$, r.t.) δ [ppm] = 7.96 (d, J = 8.3 Hz, 2H), 7.85 (d, J = 8.0 Hz, 2H), 7.80 (d, J = 8.0 Hz, 2H), 7.58 (d, J = 8.1 Hz, 2H), 7.54 (d, J = 1.6 Hz, 2H), 7.45 – 7.39 (m, 4H).

^{13}C NMR (126 MHz, $CDCl_3$, r.t.) δ [ppm] = 141.95, 140.30, 139.62, 138.29, 136.08, 129.13, 128.84, 127.62, 123.93, 121.95, 121.67, 120.22, 113.21, 93.94.

MS (MALDI-TOF pos, DCTB): m/z calculated for $C_{24}H_{14}Br_2IN$: 600.85; observed 600.9.

16

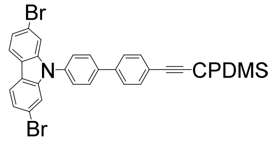

15 (300 mg, 0.5 mmol), $PdCl_2(PPh_3)_2$ (35 mg, 0.05 mmol) and CuI (19 mg, 0.1 mmol) were suspended in THF (7 mL) and NEt_3 (5 mL). CPDMS acetylene (91 mg, 0.6 mmol) was added and the suspension was stirred at r.t. for 2 h. Water and DCM were added, the organic phase was separated, washed twice with aqueous HCl (1M), once with brine, and dried over $MgSO_4$. Removal of the solvent and purification by column chromatography (Cy:DCM = 3:7, R_f = 0.55) yielded **16** (230 mg, 0.37 mmol, 74 %) as a yellow oil.

^{1}H NMR (500 MHz, $CDCl_3$, r.t.) δ [ppm] = 7.95 (d, J = 8.3 Hz, 2H), 7.83 (d, J = 8.4 Hz, 2H), 7.65 (d, J = 8.3 Hz, 2H), 7.61 (d, J = 8.3 Hz, 2H), 7.59 – 7.55 (m, 2H), 7.54 (d, J = 1.6 Hz, 2H), 7.41 (dd, J = 8.3 Hz, 1.7 Hz, 2H), 2.47 (t, J = 7.0 Hz, 2H), 1.93 – 1.83 (m, 2H), 0.92 – 0.86 (m, 2H), 0.30 (s, 6H).

^{13}C NMR (126 MHz, $CDCl_3$, r.t.) δ [ppm] = 141.9, 140.4, 140.2, 136.0, 132.8, 128.9, 127.5, 127.1, 123.9, 123.9, 122.4, 121.9, 121.7, 120.2, 119.9, 113.2, 113.2, 106.2, 93.4, 20.8, 20.6, 15.9, -1.6, - 1.7.

MS (EI, 70 eV), m/z calculated for $C_{32}H_{26}Br_2N_2Si$: 624.02; observed 624.1.

18

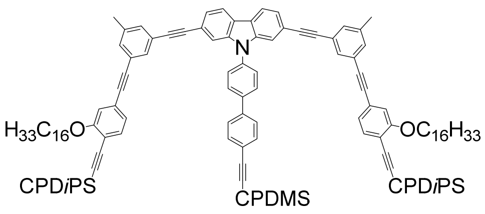

16 (590 mg, 0.94 mmol), **17** (4.60 g, 6.95 mmol), $Pd(PPh_3)_4$ (220 mg, 0.19 mmol) and CuI (73 mg, 0.38 mmol) were suspended in THF (20 mL) and piperidine (20 mL). The suspension was stirred at 80 °C for 21 h. After cooling, water and DCM were added, and the organic phase was separated and washed with aqueous HCl (1M) and brine, and dried over $MgSO_4$. Removal of the solvent and purification by column chromatography (Cy:DCM = 3:7, R_f = 0.27) yielded **18** (1.20 g, 0.67 mmol, 71 %) as a yellow viscous substance.

^{1}H NMR (500 MHz, $CDCl_3$, r.t.) δ [ppm] = 8.11 (d, *J* = 8.0 Hz, 2H), 7.90 – 7.84 (m, 2H), 7.70 – 7.64 (m, 4H), 7.63 – 7.59 (m, 4H), 7.53 (t, *J* = 1.5 Hz, 2H), 7.48 (dd, *J* = 8.1 Hz, 1.3 Hz, 2H), 7.38 (d, *J* = 7.8 Hz, 2H), 7.35 – 7.29 (m, 4H), 7.03 (dd, *J* = 7.8 Hz, 1.4 Hz, 2H), 6.97 (d, *J* = 1.4 Hz, 2H), 4.01 (t, *J* = 6.4 Hz, 4H), 2.49 – 2.42 (m, 6H), 2.35 (s, 6H), 1.94 – 1.77 (m, 12H), 1.53 – 1.45 (m, 4H), 1.38 – 1.21 (m, 52H), 1.16 – 1.03 (m, 28H), 0.89 – 0.86 (m, 6H), 0.30 (s, 6H).

^{13}C NMR (126 MHz, $CDCl_3$, r.t.) δ [ppm] = 160.1, 141.3, 140.4, 140.1, 138.5, 136.5, 133.8, 132.8, 132.4, 132.2, 131.9, 128.8, 127.7, 127.1, 124.5, 124.2, 123.7, 123.6, 123.3, 123.2, 122.3, 120.9, 120.7, 119.9, 119.8, 114.5, 113.3, 112.9, 106.3, 103.9, 95.4, 93.4, 90.9, 90.4, 89.6, 89.0, 68.7, 32.1, 29.9, 29.8, 29.6, 29.5, 29.4, 27.1, 26.3, 22.8, 21.5, 21.2, 20.9, 20.8, 20.6, 18.4, 18.1, 15.9, 14.3, 11.9, 9.8.

MS (MALDI-TOF pos, DCTB): *m/z* calculated for $C_{122}H_{150}N_4O_2Si_3$: 1787.11; observed 1787.1.

5

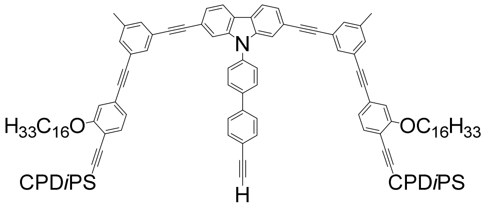

18 (1.20 g, 0.67 mmol) was dissolved in DCM (50 mL). K_2CO_3 (277,8 mg, 2.01 mmol) and MeOH (20 mL) were added, and the suspension was stirred at r.t. for 2 h. Water and DCM were added, the organic phase was separated and washed with aqueous HCl (1M), and brine, and dried over $MgSO_4$. Removal of the solvent under reduced pressure and purification by column chromatography (Cy:DCM = 1:1, R_f = 0.27) yielded **5** (0.71 g, 0.42 mmol, 63 %) as a yellow wax.

^{1}H NMR (400 MHz, $CDCl_3$, r.t.) δ [ppm] = 8.10 (d, *J* = 8.1 Hz, 2H), 7.87 (d, *J* = 8.4 Hz, 2H), 7.71 – 7.59 (m, 8H), 7.53 (t, *J* = 1.5 Hz, 2H), 7.48 (dd, *J* = 8.0 Hz, 1.3 Hz, 2H), 7.38 (d, *J* = 7.8 Hz, 2H), 7.35 – 7.33 (m, 2H), 7.32 – 7.30 (m, 2H), 7.03 (dd, *J* = 7.8 Hz, 1.4 Hz, 2H), 6.97 (d, *J* = 1.4 Hz, 2H), 4.01 (t, *J* = 6.4 Hz, 4H), 3.18

(s, 1H), 2.44 (t, J = 7.0 Hz, 4H), 2.35 (s, 6H), 1.96 – 1.76 (m, 8H), 1.54 – 1.45 (m, 4H), 1.40 – 1.21 (m, 48H), 1.18 – 1.05 (m, 28H), 0.94 – 0.78 (m, 10H).

^{13}C NMR (126 MHz, $CDCl_3$, r.t.) δ [ppm] = 160.1, 141.4, 140.5, 140.1, 138.5, 136.6, 133.8, 132.9, 132.4, 132.2, 131.9, 128.9, 127.7, 127.2, 124.5, 124.2, 123.7, 123.7, 123.3, 121.8, 121.0, 120.7, 119.9, 114.6, 113.3, 113.0, 103.9, 95.4, 90.9, 90.4, 89.6, 89.0, 83.5, 78.3, 68.7, 32.1, 29.9, 29.8, 29.6, 29.5, 29.4, 27.1, 26.3, 22.8, 21.5, 21.2, 20.9, 18.4, 18.1, 14.3, 12.0, 9.8.

MS (MALDI-TOF pos, DCTB): m/z calculated for $C_{116}H_{139}N_3O_2Si_2$: 1662.04; observed 1661.9.

3.3 Synthesis of **17**

17 was obtained by twofold Sonogashira-Hagihara reaction of TMS acetylene and CPD*i*PS acetylene utilizing the iodine/bromide selectivity. Subsequent deprotection of the TMS group and Sonogashira coupling with **22** followed by deprotection of the CPDMS protective group yielded the acetylene **17**.

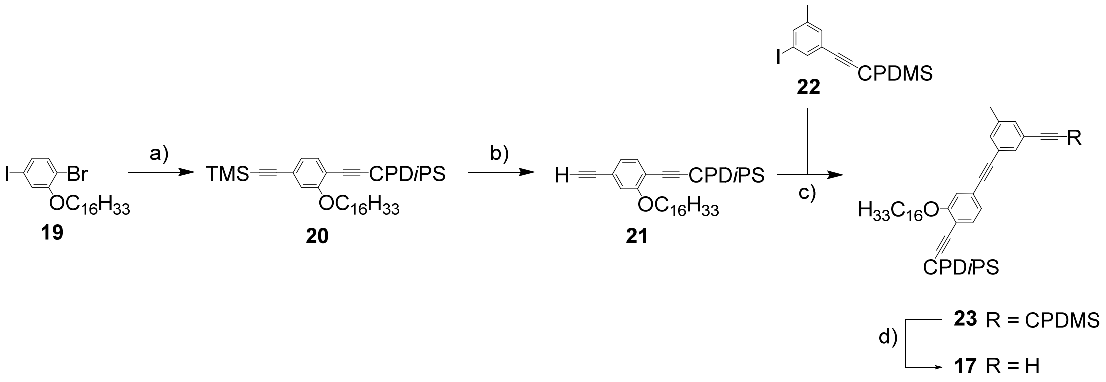

Scheme S3. a) 1.) piperidine, THF, TMS acetylene, r.t.; 20 h; 2.) CPD*i*PS acetylene, THF, 55°C, 24 h, 86 %; b) THF, MeOH, K_2CO_3, r.t., 3 h, 87 %; c) $Pd(PPh_3)_4$, CuI, THF, NEt_3, rt, 20 h, 100 %;d) K_2CO_3, MeOH, THF, r.t., 2 h, 66 %.

19

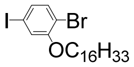

19 was synthesized according to literature procedures described ref [S3].

20

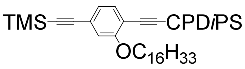

19 (8.207 g 15.68 mmol), $PdCl_2(PPh_3)_2$ (330 mg, 0.47 mmol, 3 mol%), PPh_3 (165 mg, 0.63 mmol, 4 mol%) and CuI (47 mg, 0.39 mmol, 2.5 mol%) were dissolved in piperidine (20 mL) and THF (5 mL). TMS acetylene (1.617 g, 16.46 mmol) was slowly added to the reaction mixture. After 20 h stirring at r.t., CPD*i*PS acetylene (4.88 g, 23.52 mmol) in THF (5 mL) was added slowly, and the suspension was stirred at 55 °C for 24 h. Water and DCM were added, the organic phase separated and extracted two times with water, washed with HCl (1M) and brine, and dried over Na_2SO_4. Removal of the solvent and purification by column chromatography (Cy:DCM = 2:1, R_f = 0.3) yielded **20** (8.38 g, 13.5 mmol, 86 %) as a yellow oil.

^{1}H NMR (400 MHz, $CDCl_3$, r.t.): δ [ppm] = 7.32 (d, *J* = 7.8 Hz, 1H), 6.97 (dd, *J* = 7.8 Hz, 1.4 Hz, 1H), 6.91 (d, *J* = 1.2 Hz, 1H), 3.98 (t, *J* = 6.4 Hz, 2H), 2.42 (t, *J* = 7.0 Hz, 2H), 1.96 – 1.74 (m, 4H), 1.46 (d, *J* = 7.7 Hz, 2H), 1.28 (d, *J* = 16.2 Hz, 24H), 1.14 – 1.03 (m, 14H), 0.88 (t, *J* = 6.9 Hz, 3H), 0.86 – 0.78 (m, 2H), 0.28 – 0.22 (m, 9H);

^{13}C NMR (101 MHz, $CDCl_3$): δ [ppm] = 160.0, 133.6, 124.5, 124.1, 119.9, 114.9.

MS (ESI+): *m/z* calculated for $C_{39}H_{65}NOSi_2$: 619.46; observed 619.46.

21

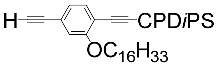

20 (500 mg, 0.81 mmol) was dissolved in THF (3 mL) and MeOH (3 mL) and K_2CO_3 (334 mg, 2.42 mmol) was added. After 3 h stirring at r.t., water and Et_2O were added, the organic phase was separated, washed with water and brine, and dried over $MgSO_4$. Removal of the solvent and purification by column chromatography (Cy:DCM = 2:1, R_f = 0.4) yielded **21** (387 mg, 0.71 mmol, 87 %) as a yellow oil.

^{1}H NMR (400 MHz, $CDCl_3$, r.t.): δ [ppm] = 7.35 (d, *J* = 7.8 Hz, 1H), 7.00 (dd, *J* = 7.8 Hz, 1.3 Hz, 1H), 6.94 (d, *J* = 1.2 Hz, 1H), 3.98 (t, *J* = 6.4 Hz, 2H), 3.14 (s, 1H), 2.42 (t, *J* = 7.0 Hz, 2H), 1.94 – 1.84 (m, 2H), 1.84 – 1.75 (m, 2H), 1.52 – 1.44 (m, 2H), 1.28 (d, *J* = 16.3 Hz, 24H), 1.15 – 1.03 (m, 14H), 0.91 – 0.85 (m, 3H), 0.85 – 0.79 (m, 2H).

^{13}C NMR (101 MHz, $CDCl_3$, r.t.): δ [ppm] = 160.0, 133.7, 124.1, 123.5, 119.9, 115.1, 113.5, 103.7, 95.5, 83.6, 78.7, 68.7, 32.1, 29.9, 29.8, 29.6, 29.5, 29.4, 27.1, 26.2, 22.8, 21.5, 20.9, 18.4, 18.1, 14.3, 11.9, 9.7

MS (EI, 70 eV, 150 °C): *m/z* calculated for $C_{36}H_{57}NOSi$: 547,42; observed 547.4.

22

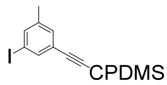

3,5-Diiodotoluene (8.08 g, 23.5 mmol), $PdCl_2(PPh_3)_2$ (0.82 g, 1.18 mmol), PPh_3 (1.23 g, 4.7 mmol) and CuI (0.45 g, 2.35 mmol) were suspended in THF (50 mL) and NEt_3 (40 mL). CPDMS acetylene (4.27 g, 28.2 mmol) was added and the suspension was stirred at 40 °C for 22 h. After cooling to r.t., DCM and water were added, the organic phase was separated, washed twice with aqueous HCl (1M), once with brine, and dried over $MgSO_4$. Removal of the solvent and purification by column chromatography (Cy:DCM = 1:1, R_f = 0.45) yielded **22** (4.63 g, 12.62 mmol, 54 %) as a yellow liquid.

^{1}H NMR (400 MHz, $CDCl_3$, r.t.) δ [ppm] = 7.64 – 7.59 (m, 1H), 7.54 – 7.49 (m, 1H), 7.25 – 7.22 (m, 1H), 2.43 (t, J = 7.0 Hz, 2H), 2.27 (s, 3H), 1.92 – 1.75 (m, 2H), 0.92 – 0.77 (m, 2H), 0.24 (s, 6H).

^{13}C NMR (101 MHz, $CDCl_3$, r.t.) δ [ppm] = 140.1, 138.7, 137.7, 132.0, 124.5, 119.8, 104.8, 93.6, 93.3, 20.9, 20.7, 20.6, 15.8.

MS (EI, 70 eV): *m/z* calculated for $C_{15}H_{18}INSi$: 367.03; observed 367.0.

23

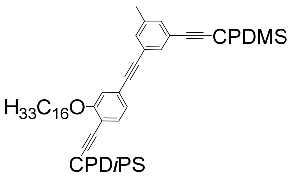

21 (5.78 g, 10.55 mmol) and **22** (3.87 g, 10.55 mmol) were dissolved in THF (20 mL). $Pd(PPh_3)_4$ (0.60 g, 0.53 mmol), CuI (0.20 mg, 1.05 mmol) and NEt_3 (20 mL) were added and the suspension was stirred at r.t. for 20 h. DCM and water were added, the organic phase was separated, washed twice with aqueous HCl (1M), once with brine and dried over $MgSO_4$. Removal of the solvent and purification by column chromatography (Cy:DCM = 1:1, R_f = 0.3) yielded **23** (8.3 g, 10.54 mmol, 100 %) as a brown oil.

^{1}H NMR (500 MHz, CD_2Cl_2, r.t.) δ [ppm] = 7.45 – 7.43 (m, 1H), 7.39 (d, J = 7.8 Hz, 1H), 7.36 – 7.32 (m, 1H), 7.30 – 7.27 (m, 1H), 7.05 (dd, J = 7.8 Hz, 1.4 Hz, 1H), 7.01 (d, J = 1.5 Hz, 1H), 4.02 (t, J = 6.3 Hz, 2H), 2.53 – 2.38 (m, 4H), 2.34 (s, 3H), 1.97 – 1.75 (m, 6H), 1.54 – 1.46 (m, 2H), 1.40 – 1.21 (m, 28H), 1.17 – 1.06 (m, 14H), 0.95 – 0.73 (m, 9H).

^{13}C NMR (126 MHz, $CDCl_3$, r.t.) δ [ppm] = 160.6, 139.3, 134.1, 133.2, 133.0, 132.5, 124.9, 124.0, 123.7, 123.6, 120.3, 115.1, 113.5, 105.9, 104.2, 96.1, 93.3, 90.5, 89.9, 69.3, 32.5, 30.3, 30.2, 30.0, 29.9, 27.5, 26.7, 23.3, 21.9, 21.3, 21.2, 21.0, 18.6, 18.3, 16.2, 14.5, 12.4, 10.2.

MS (MALDI-TOF pos, DCTB): *m/z* caclulated for $C_{51}H_{74}N_2OSi_2$: 786.53; observed 786.6.

17

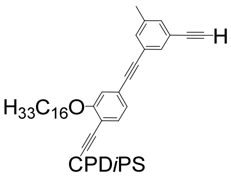

23 (8.3 g, 10.54 mmol) and K_2CO_3 (4.4 g, 31.62 mmol) were suspended in MeOH (50 mL) and THF (50 mL) and the suspension was stirred at r.t. for 2 h. DCM and water were added, the organic phase was separated, washed with brine, and dried over $MgSO_4$. Removal of the solvent under reduced pressure and purification by column chromatography (Cy:DCM = 1:1, R_f = 0.44) yielded **17** (4.6 g, 6.95 mmol, 66 %) as a colorless solid.

^{1}H NMR (500 MHz, $CDCl_3$, r.t.) δ [ppm] = 7.47 (s, 1H), 7.38 (d, *J* = 7.8 Hz, 1H), 7.33 (s, 1H), 7.28 (s, 1H), 7.03 (dd, *J* = 7.7 Hz, 1.3 Hz, 1H), 6.97 (s, 1H), 4.01 (t, *J* = 6.4 Hz, 2H), 3.06 (s, 1H), 2.48 - 2.38 (m, 2H), 2.33 (s, 3H), 1.95 – 1.76 (m, 4H), 1.53 – 1.45 (m, 2H), 1.39 – 1.21 (m, 26H), 1.19 – 1.03 (m, 14H), 0.91 – 0.84 (m, 3H).

^{13}C NMR (126 MHz, $CDCl_3$, r.t.) δ [ppm] = 160.1, 138.5, 133.8, 132.9, 132.7, 132.4, 124.4, 123.7, 123.2, 122.4, 119.9, 114.5, 113.0, 103.9, 95.4, 90.2, 89.7, 83.0, 77.6, 68.7, 32.1, 29.9, 29.8, 29.6, 29.5, 29.4, 27.1, 26.3, 22.8, 21.5, 21.2, 20.9, 18.4, 18.1, 14.3, 11.9, 9.7.

MS (MALDI-TOF pos, DCTB): *m/z* calculated for $C_{45}H_{63}NOSi$: 661.47; observed 660.2.

3.4 Synthesis of **6a** and **6b**

Fourfold iodination of tetraphenylmethane in para position followed by a statistical Sonogashira reaction with propargyl alcohol lead to the central unit **6a**, while coupling with **26** lead to **6b**, respectively.

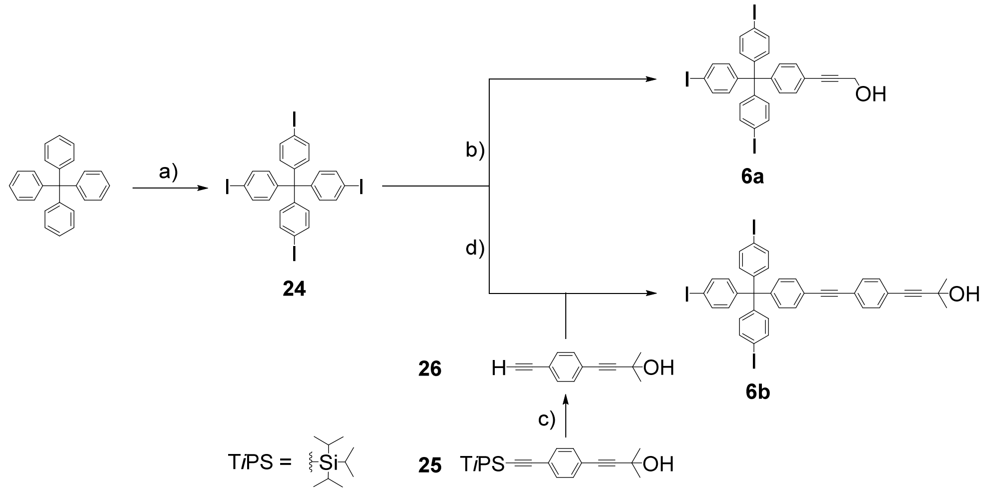

Scheme S4. a) I_2, $PhI(O_2CCF_3)_2$, 80 °C, 21 h, 60 %; b) propargyl alcohol, $Pd(PPh_3)_4$, PPh_3, CuI, THF, piperidine, 50 °C, 22 h, 25 %; c) TBAF, THF, DCM, r.t., 20 h, 95 %; d) $PdCl_2(PPh_3)_2$, CuI, PPh_3, THF, piperidine, 40 °C, 18 h, 24 %.

24

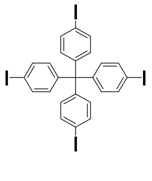

Tetraphenylmethane (580 mg, 1.81 mmol) was dissolved in $CHCl_3$ (15 mL). I_2 (1.38 g, 4.53 mmol) and $PhI(O_2CCF_3)_2$ (1.38 g, 5.43 mmol) were added and the suspension was stirred at 80 °C for 21 h. After cooling to r.t., the precipitate was filtered off and washed with DCM. The product was dried under vacuum and yielded **24** (900 mg, 1.09 mmol, 60 %) as a colorless solid.

^{1}H NMR (400 MHz, $CDCl_3$, r.t.) δ [ppm] = 7.58 (d, *J* = 8.6 Hz, 8H), 6.88 (d, *J* = 8.7 Hz, 8H).

^{13}C NMR (125 MHz, $CDCl_3$, 298 K) δ [ppm] = 145.08 (C-2), 137.10 (C-4), 132.63 (C- 3).

MS (EI, 70 eV): *m/z* calculated for $C_{25}H_{16}I_4$: 823.74; observed 823.7.

6a

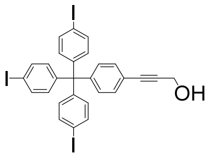

24 (2.1 g, 2.55 mmol), $PdCl_2(PPh_3)_2$ (179 mg, 0.26 mmol), PPh_3 (270 mg, 1.02 mmol) and CuI (97 mg, 0.51 mmol) were suspended in THF (100 mL) and piperidine (40 mL). Propargyl alcohol (286 mg, 5.1 mmol) was added, and the suspension was stirred for 22 h at 50 °C. After cooling to r.t., the suspension was diluted with water and DCM, the organic phase was separated, washed three times with aqueous HCl (1M), once with brine, and dried over $MgSO_4$. Removal of the solvent and purification by column chromatography (DCM, R_f = 0.6) yielded **6a** (488 mg, 0.65 mmol, 25 %) as a colorless solid.

^{1}H NMR (500 MHz, $CDCl_3$, r.t.) δ [ppm] = 7.60 – 7.55 (m, 6H), 7.33 (d, J = 8.5 Hz, 2H), 7.09 (d, J = 8.5 Hz, 2H), 6.90 – 6.86 (m, 6H), 4.48 (s, 2H).

^{13}C NMR (126 MHz, $CDCl_3$, r.t.) δ [ppm] = 145.8, 145.3, 137.2, 132.8, 131.4, 130.7, 120.9, 92.6, 87.9, 85.3, 64.3, 51.8.

MS (MALDI-TOF pos, DCTB): *m/z* calculated for $C_{28}H_{19}I_3O$: 751.86; observed 751.8.

25

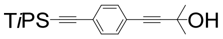

1,4-Diiodobenzene (1.50 g, 4.55 mmol), $PdCl_2(PPh_3)_2$ (160 mg, 0.228 mmol), CuI (86.7 mg, 0.455 mmol) and PPh_3 (59.8 mg, 0.228 mmol) were suspended in THF (20 mL) and piperidine (5 mL). 2-Methylbut-3-yn-2-ol (383 mg, 4.55 mmol) was added and the suspension was stirred at 40 °C for 3 h. T*i*PS acetylene (996 g, 5.46 mmol) was added and the suspension was stirred at 40 °C for another 17 h. Water and DCM were added, the organic phase was separated and washed twice with brine, and dried over Na_2SO_4. Removal of the solvent and purification by column chromatography (DCM, R_f = 0.5), yielded **25** (759 mg, 2.23 mmol, 49 %) as a brown-yellow oil.

^{1}H NMR (500 MHz, $CDCl_3$, r.t.): δ [ppm] = 7.40z (d, J = 8.5 Hz, 2H), 7.34 (d, J = 8.5 Hz, 2H), 1.62 (s, 6H), 1.12 (s, 21H).

^{13}C NMR (126 MHz, $CDCl_3$, r.t.) δ [ppm] = 132.01, 131.56, 123.52, 122.73, 106.68, 95.59, 92.76, 82.00, 65.80, 31.58, 18.80, 11.44.

MS (EI, 70 eV): *m/z* calculated for $C_{22}H_{32}OSi$: 340.22; observed 340.2.

26

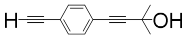

25 (822 mg, 2.41 mmol) was dissolved in DCM (30 mL). TBAF (6.03 mL, 6.03 mmol, 1M in THF) was added and the solution was stirred at r.t. for 20 h. Water was added, the organic phase was separated and washed twice with brine, and dried over Na_2SO_4. The solvent was removed under reduced pressure. After column chromatographic purification on silica gel (DCM, $R_f = 0.3$), **26** (423 mg, 2.30 mmol, 95 %) was obtained as a yellow solid.

^{1}H NMR (500 MHz, $CDCl_3$, r.t.): δ [ppm] = 7.42 (d, J = 8.5 Hz, 2H), 7.35 (d, J = 8.5 Hz, 2H), 3.15 (s, 1H), 1.61 (s, 6H).

^{13}C NMR (126 MHz, $CDCl_3$, r.t.) δ [ppm] = 132.1, 131.7, 123.4, 122.1, 95.9, 83.3, 81.8, 79.0, 65.8, 31.6

MS (EI, 70 eV): *m/z* calculated for $C_{13}H_{12}O$: 184.09; observed 184.1.

6b

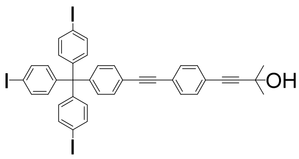

24 (1.24 mg, 1.51 mmol), $PdCl_2(PPh_3)_2$ (175 mg, 0.151 mmol), CuI (57.52 mg, 0.302 mmol) and PPh_3 (158 mg, 0.604 mmol) were suspended in THF (40 mL) and piperidine (25 mL). **26** (416 mg, 2.26 mmol) was added and the suspension was stirred at 40 °C for 18 h. Water and DCM were added, the organic phase was separated and the aqueous phase was extracted twice with DCM. The combined organic phase was washed twice with aqueous HCl (1M), once with brine, and dried over Na_2SO_4. The solvent was removed under reduced pressure. After column chromatographic purification on silica gel (DCM, $R_f = 0.39$), **6b** (355 mg, 0.404 mmol, 24 %) was obtained as a white solid.

^{1}H NMR (500 MHz, $CDCl_3$, r.t.): δ [ppm] = 7.59 (d, J = 8.8 Hz, 6H), 7.47–7.35 (m, 6H), 7.13 (d, J = 8.6 Hz, 2H), 6.90 (d, J = 8.8 Hz, 6H), 1.62 (s, 6H).

^{13}C NMR (126 MHz, $CDCl_3$, r.t.) δ [ppm] = 145.3, 137.2, 132.8, 131.7, 131.6, 131.3, 130.8, 123.1, 122.9, 121.5, 92.6, 65.8, 64.4, 31.6.

MS (MALDI-TOF pos, DCTB): *m/z* calculated for $C_{38}H_{27}I_3O$: 879.92; observed 879.92.

3.5 Synthesis of **11**

27 was synthesized by reductive amination of 7-tridecanone. Condensation with perylene-3,4,9,10-tetracarboxylic dianhydride and subsequent partial hydrolysis of the resulting bisimide **28** under basic conditions yielded **29**. Decarbonylation and decarboxylation using copper bronze gave the perylene monoimide **30**, that was iodinated to give **11**.

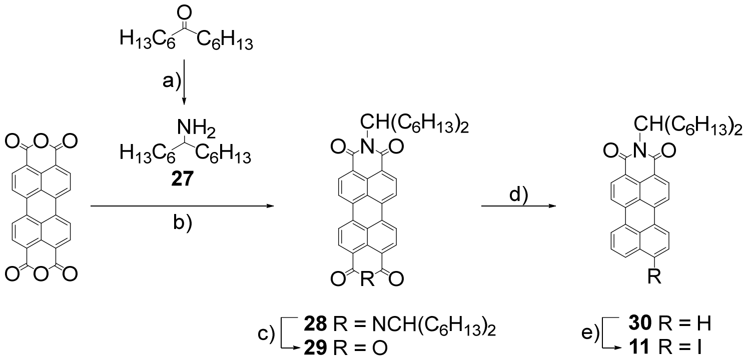

Scheme S5: a) Ammonium acetate, MeOH, $NaBH_3CN$,r.t., 90 h, 98 %; b) imidazole, 180 °C, 5 h, 89 %; c) KOH, ^{t}BuOH, 100 °C, 45 min, 48 %; d) Cu bronze, 3-methylpyridine, 175 °C, 24 h, 43 %, e) I_2, H_5IO_6, HOAc, H_2SO_4, $CHCl_3$, 85 °C, 24 h, 30 %.

27

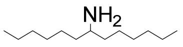

7-Tridecanone (2.50 g, 12.6 mmol) and dried ammonium acetate (10.0 g, 129 mmol) were suspended in MeOH (38 mL). The reaction mixture was heated until all components were dissolved. The mixture was allowed to cool to r.t. and $NaBH_3CN$ (0.56 g, 8.91 mmol) was added and the reaction mixture was stirred at r.t. for 90 h. After cooling the reaction mixture to 0 °C, aq. HCl (37 %) was added dropwise until a colorless precipitate was observed (at pH ~ 7). The precipitate was filtered off, and was then dispersed in water (200 mL). Aq. KOH solution (1M) was added until pH ~ 10 was reached. The aqueous solution was extracted two times with $CHCl_3$, and the combined organic phase was dried over Na_2SO_4. Removal of the solvent under reduced pressure yielded pure **27** (2,47 g, 12.4 mmol, 98 %) as a yellowish oil.

Analytical data coincide with those in ref [S4].

28

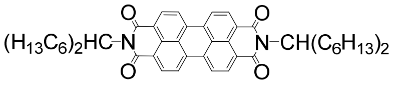

Perylene-3,4,9,10-tetracarboxylic dianhydride (1,28 g, 3.27 mmol), **27** (1.60 g, 8.02 mmol) and imidazole (5.22 g) were stirred at 180 °C for 5 h. After cooling to r.t., EtOH was (85 mL) added. The residue was filtered off and was then stirred in aq. HCl (2 M, 100 mL) at r.t for 14 h. After filtration, the solid was washed with water until pH = 7 was reached and subsequently dried at 130 °C yielding pure **28** (2.21 g, 2,93 mmol, 89 %) as a red solid.

Analytical data coincide with those in ref [S4].

29

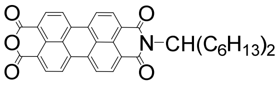

28 (755 mg, 1.00 mmol) was suspended and stirred in ^{t}BuOH (12 mL) at 100 °C. KOH (188 mg, 3.35 mmol) was dissolved in ^{t}BuOH (6 mL) in the heat and was added to the reaction mixture. After 45 min, the reaction mixture was cooled to r.t.. Glacial acid (17 mL) and aq. HCl (2 M, 8 mL) were added and the mixture was stirred at r.t. for another 14 h. The dark red solid was filtered off, washed with water until pH = 7 was reached and dried under vacuum. Purification *via* column chromatography (DCM→DCM:AcOH = 95:5, R_f = 0.6 (DCM:AcOH = 95:5)) yielded pure **29** (274 mg, 0.48 mmol, 48 %) as a red solid.

Analytical data coincide with those in ref [S4].

30

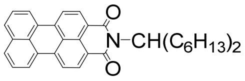

Copper powder (2.46 g, 38.71 mmol) was activated at 80 °C for 24 h in 3-picoline (16 mL) under moisture exclusion (drying tube, $CaCl_2$). **29** (2.16 g, 3.77 mmol) was added to the reaction mixture and stirred at 175 °C for 24 h. After cooling to r.t., $CHCl_3$ and aq. HCl (3.2 M) were added. The aqueous phase was extracted two times with $CHCl_3$. The combined organic phase was washed with water and dried over Na_2SO_4. Removal of the solvent and purification *via* column chromatography (DCM:PE = 2:1, R_f = 0.6) yielded pure **30** (818 mg, 1.62 mmol, 43 %) as a red solid.

Analytical data coincide with those in ref [S5].

11

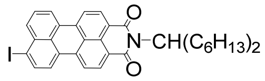

30 (630 mg, 1.25 mmol), iodine (150 mg, 1.18 mmol) and H_5IO_6 (143 mg, 0.627 mmol) were suspended in glacial acid (1 mL), 30% sulfuric acid (0.2 mL) and $CHCl_3$ (0.7 mL). The mixture was stirred at 85 °C for 24 h. Afterwards iodine (164 mg, 1.29 mmol) and H_5IO_6 (143 mg, 0.627 mmol), glacial acid (1 mL), 30% sulfuric acid (0.2 mL) and $CHCl_3$ (0.7 mL) were added again. The mixture was also stirred at 85 °C for 24 h. After cooling to r.t., $CHCl_3$ (100 mL) was added, and the reaction was quenched by adding 30% aq. $NaHSO_3$ solution (100 mL). The aqueous phase was extracted two times with $CHCl_3$. The combined organic phase was dried over Na_2SO_4. Removal of the solvent and purification *via* column chromatography (DCM:PE = 2:1, R_f = 0.4) yielded pure **11** (0.233 g, 0.37 mmol, 30 %) as a red solid.

Analytical data coincide with those in ref [S6].

3.6 Synthesis of **4**

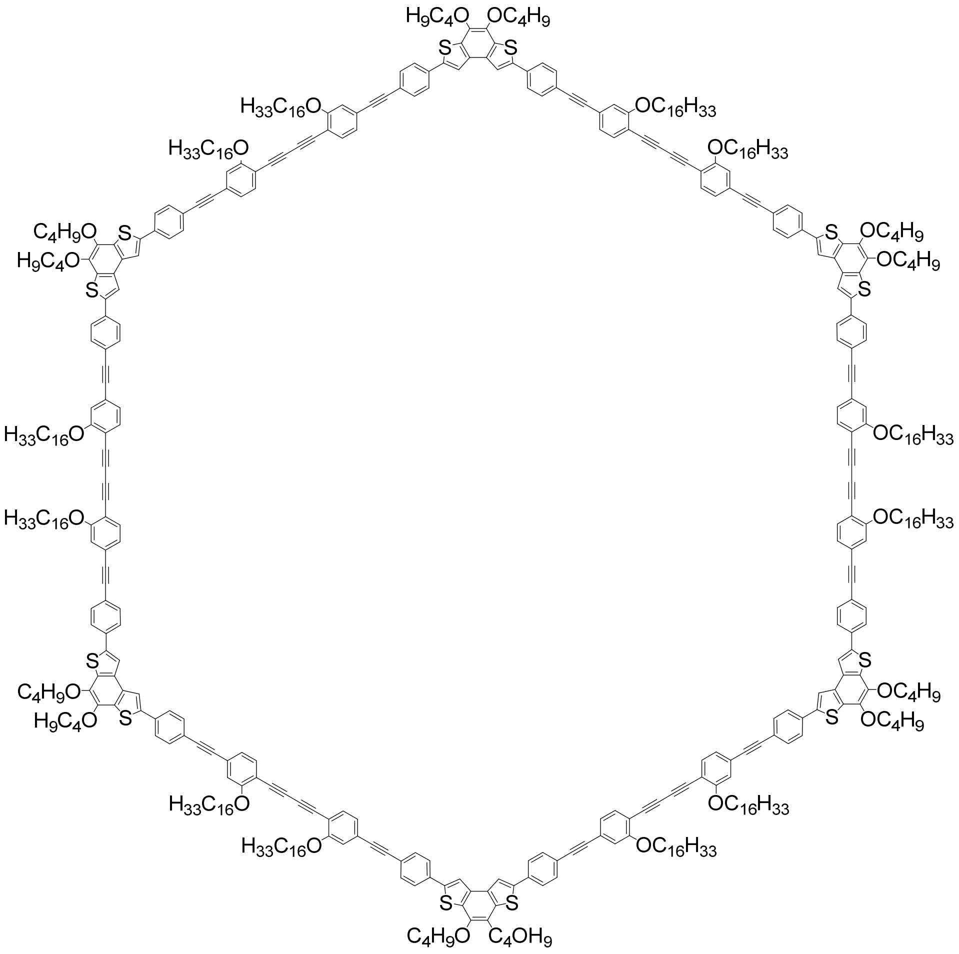

4 was synthesized according to ref [S7].

4 References

[S1] a) S. Höger, K. Bonrad, *J. Org. Chem.* **2000**, *65*, 2243-2245; b) G. Gaefke, S. Höger, *Synthesis* **2008**, *14*, 2155-2157.

[S2] T. R. Rusch, M.Hammerich, R. Herges, O. M. Magnussen, *Chem. Commun.* **2019**, *55*, 9511-9514.

[S3] T. J. Keller, J. Bahr, K. Gratzfeld, N. Schönfelder, M. A. Majewski, M. Stępień, S. Höger, S.-S. Jester, *Beilstein J. Org. Chem.* **2019**, *15*, 1848-1855.

[S4] M. W. Holman, R. Liu, D. M. Adams, *J. Am. Chem. Soc.* **2003**, *125*, 12649-12654.

[S5] L. Feiler, H. Langhals, K. Polborn, *Liebigs Ann.* **1995**, 1229-1244.

[S6] H. Langhals, J. Büttner, P. Blanke, *Synthesis* **2005**, *3*, 364-366.

[S7] S.-S. Jester, E. Sigmund, S. Höger, *J. Am. Chem. Soc.* **2011**, *133*, 11062-11065.
